# Discovery of A Novel Series of Quinazoline–Thiazole Hybrids as Potential Antiproliferative and Anti-Angiogenic Agents

**DOI:** 10.3390/biom14020218

**Published:** 2024-02-12

**Authors:** Alexandru Șandor, Ionel Fizeșan, Ioana Ionuț, Gabriel Marc, Cristina Moldovan, Ilioara Oniga, Adrian Pîrnău, Laurian Vlase, Andreea-Elena Petru, Ioana Macasoi, Ovidiu Oniga

**Affiliations:** 1 Department of Pharmaceutical Chemistry, Faculty of Pharmacy, “Iuliu Hațieganu” University of Medicine and Pharmacy, 41 Victor Babes, Street, 400010 Cluj-Napoca, Romania; alexandru.sandor@elearn.umfcluj.ro (A.Ș.); ioana.ionut@umfcluj.ro (I.I.); marc.gabriel@umfcluj.ro (G.M.); cmoldovan@umfcluj.ro (C.M.); ooniga@umfcluj.ro (O.O.); 2 Department of Toxicology, Faculty of Pharmacy, “Iuliu Hațieganu” University of Medicine and Pharmacy, 8 Victor Babeș Street, 400012 Cluj-Napoca, Romania; andreea.elen.petru@elearn.umfcluj.ro; 3 Department of Pharmacognosy, “Iuliu Hațieganu” University of Medicine and Pharmacy, 12 Ion Creangă Street, 400010 Cluj-Napoca, Romania; ioniga@umfcluj.ro; 4 National Institute for Research and Development of Isotopic and Molecular Technologies, 67-103 Donath Street, 400293 Cluj-Napoca, Romania; apirnau@itim-cj.ro; 5 Department of Pharmaceutical Technology and Biopharmaceutics, “Iuliu Hațieganu” University of Medicine and Pharmacy, 41 Victor Babeș, Street, 400012 Cluj-Napoca, Romania; laurian.vlase@umfcluj.ro; 6 Department of Toxicology, Faculty of Pharmacy, “Victor Babeș” University of Medicine and Pharmacy Timișoara, Eftimie Murgu Square No. 2, 300041 Timișoara, Romania; macasoi.ioana@umft.ro; 7 Research Center for Pharmaco-Toxicological Evaluations, Faculty of Pharmacy, “Victor Babes” University of Medicine and Pharmacy Timișoara, Eftimie Murgu Square No. 2, 300041 Timișoara, Romania

**Keywords:** quinazoline, thiazole, VEGFR2, structure design, synthesis, anti-angiogenic, antiproliferative, hybrid compounds, cancer, drug discovery, small molecules, tyrosine kinase inhibitors

## Abstract

Considering the pivotal role of angiogenesis in solid tumor progression, we developed a novel series of quinazoline–thiazole hybrids (**SA01–SA07**) as antiproliferative and anti-angiogenic agents. Four out of the seven compounds displayed superior antiproliferative activity (IC_50_ =1.83-4.24 µM) on HepG2 cells compared to **sorafenib** (IC_50_ = 6.28 µM). The affinity towards the VEGFR2 kinase domain was assessed through in silico prediction by molecular docking, molecular dynamics studies, and MM-PBSA. The series displayed a high degree of similarity to **sorafenib** regarding the binding pose within the active site of VEGFR2, with a different orientation of the 4-substituted-thiazole moieties in the allosteric pocket. Molecular dynamics and MM-PBSA evaluations identified **SA05** as the hybrid forming the most stable complex with VEGFR2 compared to **sorafenib**. The impact of the compounds on vascular cell proliferation was assessed on EA.hy926 cells. Six compounds (**SA01–SA05, SA07**) displayed superior anti-proliferative activity (IC_50_ = 0.79–5.85 µM) compared to **sorafenib** (IC_50_ = 6.62 µM). The toxicity was evaluated on BJ cells. Further studies of the anti-angiogenic effect of the most promising compounds, **SA04** and **SA05,** through the assessment of impact on EA.hy296 motility using a wound healing assay and in ovo potential in a CAM assay compared to **sorafenib**, led to the confirmation of the anti-angiogenic potential.

## 1. Introduction

Tumor cells are defined by a set of complex functional capabilities that are mandatory for their proliferation and growth into malignant tumors. The shared commonalities of all the various types of tumor cells, given the genetic and histological heterogeneity, differentiate them from the somatic cells and are defined as the “hallmarks of cancer”. Malignant cells have sustained proliferative signaling and can enable replicative immortality while avoiding growth suppression and cell death, ultimately leading to tumor mass build-up and metastasis [1,2,3]. Sustained survival, proliferation, and migration of the tumor cells are dependent on the oxygen and nutrient supplies that are provided by the sprouting of new blood vessels through angiogenesis [4,5]. 

Tumor neovascularization is a complex process, that is triggered by hypoxic conditions and is vital for any solid tumor to ensure the metabolic needs that facilitate expansion and metastasis [5,6]. Angiogenesis involves the loss of balance between the pro- and anti-angiogenic factors of the vascular homeostasis that leads to the rapid proliferation of the dormant endothelial cells and the development of novel blood vessels through splitting or sprouting from the already existing blood vessels [7,8]. Tumor vessels are often associated with an imperfect architecture, with large pores in their walls and increased permeability due to the defective binding between the endothelial cells [9,10]. The rapid proliferation of the endothelial cells is induced by a variety of pro-angiogenic growth factors such as vascular endothelial growth factor (VEGF), platelet-derived growth factor (PDGF), fibroblast growth factor-2 (FGF-2), angiopoietins, and chemokines that are frequently produced simultaneously and ultimately lead to the activation of several cellular signaling pathways [4,7]. VEGF is the most critical pro-angiogenic factor that is actively produced by tumor cells and stroma cells in hypoxic conditions, and it leads to the activation of several tyrosine kinase receptors including VEGF-receptor 2 (VEGFR2) [8]. During tumor neovascularization, VEGFR2 mediates endothelial cell proliferation, migration, and invasion, acting as a major cellular signaling transducer that facilitates the phosphorylation of protein kinase B (Akt), extracellular signal-regulated kinase 1 and 2 (Erk 1 and 2), mammalian target of rapamycin (mTOR), and focal adhesion kinase (FAK) and also the activation of the downstream signaling mediated by them [4,11]. 

**Figure 1:**
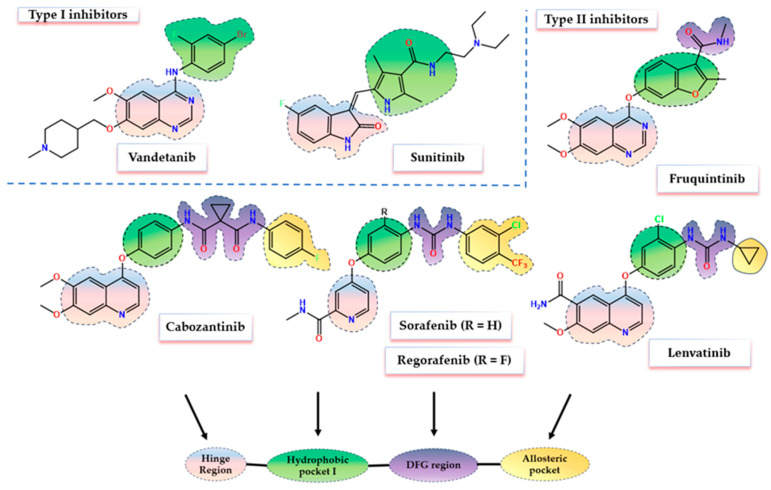
Chemical structures of the clinically approved VEGFR2 inhibitors and the distinctive structural features of the compounds in correlation with occupied regions of the catalytic cleft.

This makes VEGFR2 one of the most important targets for the development of novel new-generation anti-angiogenic therapies [12]. The discovery of small molecule inhibitors of VEGFR2 signaling represented a breakthrough in the fight against cancer [13]. In the last few years, several VEGFR2 inhibitors with distinctive structural features have been approved for clinical use, including type I inhibitors (vandetanib, sunitinib) and type II inhibitors (sorafenib, regorafenib, cabozantinib, lenvatinib, fruquintinib) (Figure 1) [14,15]. VEGFR2 small molecule inhibitors are successfully used on a grand scale for the treatment of several forms of cancer including hepatocellular carcinoma (HCC) [16], metastatic renal carcinoma [17], thyroid cancer [18], gastrointestinal stromal tumor, melanoma, and colorectal cancer [19,20,21]. The major drawbacks of the current VEGFR2 inhibition therapy are (I) the reduced selectivity profile of the compounds as most of the approved molecules act as pan-inhibitors on several other tyrosine or serine/threonine kinases leading to various undesired effects, and (II) the adaptative development of the resistance phenomenon [22,23,24,25]. 

Therefore, the purpose of this work was to develop a novel series of quinazoline derivatives as potential anti-angiogenic agents, considering the structural model of the already established VEGFR2 tyrosine kinase inhibitors (TKIs) used in therapy (Figure 1).

## 2. Structural Design and Rationale

An in-depth analysis of the X-ray crystallographic data of various VEGFR2 complexes provided by the Protein Data Base (PDB) [26], outlines the four distinctive sections that form the catalytic cleft of the receptor that comes in contact with type II inhibitors: (I) the hinge region formed by amino acids such as Cys919, Leu840, Phe918, and Glu917; (II) hydrophobic pocket I surrounded by Lys868 and the gatekeeper Val916 (gatekeeper region); (III) the DFG-motif area with the Glu885–Asp1047 pair responsible to bind the triphosphate group of the ATP and to facilitate γ-phosphorylation; and IV) the allosteric pocket formed by the outwards flip of Phe1047 of the DFG-motif (hydrophobic pocket II) [27,28,29] (Figure 1). The allosteric pocket is highly lipophilic and can provide additional selectivity due to the less conserved character of the amino acids that surround it among the human kinome [30,31]. This pocket is specifically targeted by type II inhibitors, which usually incorporate a highly lipophilic moiety capable of forming hydrophobic interactions within this region [31].

The common pharmacophores shared by the type II VEGFR2 inhibitors include four main structural elements as follows: (I) a flat aromatic nitrogen-containing heterocyclic core capable of forming stable hydrogen bonds (H-Bonds) with key amino acids (Cys919, Glu917) and hydrophobic interactions within the hinge region of the ATP-binding pocket (adenine binding region); (II) a hydrophobic linker between the main aromatic core and DFG-motif region of the kinase domain that consists of a monocyclic aromatic ring forming a suitable angle with the main core that accommodates the gatekeeper region; (III) a 3–5 atoms long hydrophilic group (urea, thiourea, amide) capable of forming both donor and acceptor H-bonds with the Glu885–Asp1047 pair; IV) a hydrophobic moiety capable of accommodating the allosteric pocket formed in the DFG-out conformation of the inactive receptor [27,28,29,32,33]. 

**Figure 2:**
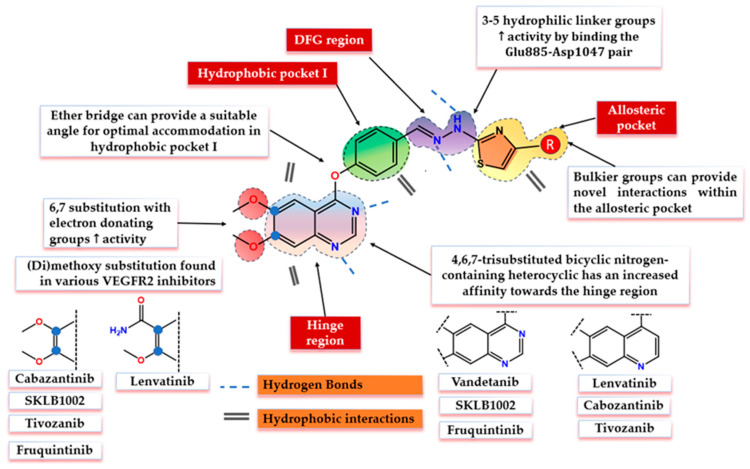
Structural design by scaffold hopping of the thiazole–quinazoline hybrid series SA01–SA07.

Motivated by the existing data, the scaffold hopping approach was applied to synthesize the series **SA01–SA07**, through the bio-isosteric replacement of the four main structural features of various type II VEGFR2 inhibitors, to elevate the antiproliferative and anti-angiogenic effect as depicted in Figure 2. 

Quinazoline was chosen as the main core due to its distinctive features and the already established capability to bind the hinge region through both H-bonds and hydrophobic interactions as reported by previous works [33,34]. Further substitution with methoxy groups in positions 6 and 7 of the core is a common modulation among the VEGFR2 inhibitors that can increase both the electronic density on the ring (though an electron-donating effect) and can contribute to the overall increase in lipophilicity. The insertion of the 4-phenoxy group on the quinazoline core can provide a suitable angle between the two aromatic moieties for a favorable biological effect and form favorable hydrophobic interactions in the gatekeeper region [26,27,28]. To maximize the affinity for the DFG-motif section, a hydrazone moiety was chosen due to its distinctive electronic features that might facilitate H-bond interactions [35]. Finally, various substituted thiazole groups were chosen to accommodate the allosteric binding pocket due to the collective observations of various research groups regarding the fact that the allosteric binding pocket can accommodate larger hydrophobic groups [26,27,28]. Also, Hassan et al. concluded that the phenyl-thiazole moieties are suitable for allosteric pocket accommodation, and they can provide a different binding mode and novel interactions compared to the typical monocyclic moieties [36]. 

## 3. Materials and Methods

### 3.1. Materials and Measurements

All reagents and solvents at analytical grade purity were acquired from local suppliers and used without any further purification according to the manufacturer’s instructions. The progress of the reaction was monitored by thin-layer chromatography (TLC) with silica gel-coated F256 (Merck) as stationary phase and ethyl acetate/heptane (7:3) as mobile phase. The uncorrected melting point (°C) values were determined by the glass capillary method using a melting point device MPM-H1 (Schorpp Gerätetechnik, Überlingen, Germany). The infrared (IR) spectra were recorded using an FT/IR 6100 spectrometer (Jasco, Cremella, Italy) in KBr pellets. The MS spectra were recorded using an Agilent 1100 series device in the positive ionization mode (Agilent Technologies, Santa Clara, CA, USA). The ^1^H-NMR (500 MHz) and ^13^C-NMR (125 MHz) spectra were recorded with an Avance NMR spectrometer (Bruker, Karlsruhe, Germany) in δ scale (ppm) using dimethylsulfoxide-*d*_6_ (DMSO-*d*_6_) as solvent. Tetramethylsilane (TMS) served as the calibration standard for the NMR spectrometer, while the solvent peak was taken as reference for determining the chemical shift values. The splitting patterns for the identified signals were abbreviated as singlet (s), doublet (d), double doublet (dd), triplet (t), multiplet (m), and broad (br). For easier tracking of the proton and carbon signals, the major molecular regions were abbreviated as follows: quinazoline (Q), the proximal phenyl moiety (Ar^1^), thiazole (Th), and the distal phenyl moiety in position 4 of the thiazole ring (Ar^2^). 

All the following cell culture reagents were purchased from Gibco (Paisley, UK): Dulbecco’s modified Eagle’s medium (DMEM) with low and high glucose, Minimum Essential Medium (MEM), fetal bovine serum (FBS), penicillin/streptomycin (10,000 U/mL), phosphate-buffered saline (PBS), Dulbecco’s phosphate-buffered saline with magnesium and calcium (D-PBS), trypsin-EDTA and sodium pyruvate (100 mM).

### 3.2. Chemistry

#### 3.2.1. Synthesis of Intermediate (1)

In a round-bottom flask, 30 mL of acetonitrile (MeCN) was added to 1.831 g of 4-hydroxy-benzaldehyde (15 mmol) and 4.146 g of anhydrous potassium carbonate (30 mmol). After the mixture was refluxed for 2 h, 3.369 g of 6,7-dimethoxy-4-chloro-quinazoline (15 mmol) was added and the reflux was continued for 10 more hours. After the completion of the reaction (monitored by TLC), the flask was left at room temperature for 1 h and then poured on crushed ice. The resulting solid was filtered, dried, and recrystallized from hot ethanol to yield the corresponding intermediate **(1)**. 

*4-((6,7-dimethoxyquinazolin-4-yl)-oxy)-benzaldehyde* (**1**): white needle crystals; mp = 167–168 °C; yield = 92.8%; FT **IR** (KBr) ν_max_ cm^−1^: 1702.28 (C=O), 1474.8 (C-N), 1569.29 (C=C), 1618.47(C=N), 2744.21–2794.33 (C-H, CH=O), 1216.38 (C-O, OCH_3_); **MS**: (M_w_= 310.10), *m/z* = 311.2 [M + H]^+^; **^1^H-NMR** (DMSO-*d*_6_, 500 MHz) *δ* ppm: 3.988 (s, 3H, Q_6_-OCH_3_), 4.006 (s, 3H, Q_7_-OCH_3_), 7.417 (s, 1H, Q-H_8_), 7.578–7.595 (m, 3H – 2H Ar^1^-H_3_, 1H Q-H_5_), 8.056 (d, 2H, Ar^1^-H_2_, *J* = 9 Hz), 8.588 (s, 1H, Q-H_2_), 10.054 (s, 1H, CH=O); **^13^C-NMR** (DMSO-*d*_6_, 125 MHz) *δ* ppm: 56.035 (Q_7_-OCH_3_), 56.196 (Q_6_-OCH_3_), 100.600 (Q_8_), 106.752 (Q_4a_), 109.727 (Q_5_), 122.927 (Ar^1^-C_3_), 131.236 (Ar^1^-C_2_), 133.626 (Ar^1^-C_1_), 149.112 (Q_6_), 152.051 (Q_7_), 155.943 (Q_4_), 157.217 (Ar^1^-C_4_), 164.286 (Q_2_), 191.989 (CH=O).

#### 3.2.2. Synthesis of Intermediate (2)

The synthesis of the target compound (**2**) was achieved by using a modified protocol previously reported [35]. In a round-bottom flask, 3.103 g of intermediate (**1**) (10 mmol) was dissolved in ethanol (25 mL) under reflux. To the reaction mixture, 1.822 g of thiosemicarbazide (20 mmol) and 1 drop of concentrated H_2_SO_4_ were added and this was left on reflux for 20 hours until completion of the reaction (monitored by TLC); a pale yellow precipitate was formed. The hot suspension was filtered under vacuum and rinsed with water to remove the thiosemicarbazide excess. The product was dried and crystallized from hot ethanol to yield the corresponding intermediate **(2)**. 

*(E)-2-(4-((6,7-dimethoxyquinazolin-4-yl-oxy)-benzylidene)-hydrazine-1-carbothioamide* (**2**): pale yellow powder, mp = 179–280 °C; yield = 85.5%; FT **IR** (KBr) ν_max_ cm^−1^: 1461.78 (C-N), 1578.45 (C=C), 1617.5 (C=N), 1213.01 (C-O, OCH_3_), 3251.88 (N-H, -NH-); **MS**: *m/z* = 384.2 [M + H]^+^; **^1^H-NMR** (DMSO-*d*_6_, 500 MHz) *δ* ppm: 3.985 (s, 3H, Q_6_-OCH_3_), 4.001 (s, 3H, Q_7_-OCH_3_), 7.372 (d, 2H, Ar^1^-H_3_, *J* = 9 Hz), 7.404 (s, 1H, Q-H_8_), 7.575 (s, 1H, Q-H_5_), 7.935 (d, 2H, Ar^1^-H_2_, *J* = 9 Hz), 8.049 (br. S, 1H, -NH_2_), 8.107 (s, 1H, -CH=N-), 8.213 (br. S, 1H, -NH_2_), 8.571 (s, 1H, Q-H_2_) 11.465 (br. S, 1H, -NH-); **^13^C-NMR** (DMSO-*d*_6_, 125 MHz) *δ* ppm: 56.014 (Q_7_-OCH_3_), 56.168 (Q_6_-OCH_3_), 100.684 (Q_8_), 106.745 (Q_4a_), 109.741 (Q_5_), 122.444 (Ar^1^-C_3_), 128.604 (Ar^1^-C_2_), 131.690 (Ar^1^-C_1_), 141.363 (-CH=N-), 148.944 (Q_8a_), 150.120 (Q_6_), 152.184 (Q_7_), 153.542 (Ar^1^-C_4_), 155.810 (Q_4_), 164.608 (Q_2_), 177.970 (C=S).

#### 3.2.3. Synthesis of Compounds SA01–SA07

The synthesis of the target compounds was achieved by using a modified previously reported protocol [35]. A quantity of 200 mg of intermediate (**2**) (0.52 mmol) was mixed with 10 mL of a hot acetone/DMF (10:1) mixture and stirred at room temperature for 1 h, until dissolution. To the obtained solution, 0.52 mmol of the corresponding α-haloketones was added and the stirring continued at room temperature for 8 more hours for compounds **SA01**, **SA03-SA05,** and **SA07** and for 8 h at room temperature and 2 h under reflux for compound **SA02**. The precipitate formed for each compound was filtered off, dried under vacuum, and washed with KHCO_3_ solution until a neutral pH was reached. The compounds were crystallized from acetone/DMF to afford the corresponding compounds **SA01-SA05,** and **SA07**.

A different protocol was applied for the synthesis of **SA06**. A quantity of 200 mg of compound 2 (0.52 mmol) was mixed with 10 mL of acetone and refluxed for 30 min until dissolution. A total of 50 mg (0.54 mmol) of chloroacetone was added dropwise to the mixture and this was left on reflux for 4 h until the completion of the reaction (monitored by TLC). The pale yellow precipitate was isolated using the same protocol previously reported for compounds **SA01-SA05** and **SA07**, yielding the corresponding compound **SA06**.

*(E)-2-(2-(4-((6,7-dimethoxyquinazolin-4-yl)oxy)-benzylidene)hydrazineyl)-4-(4-methoxyphenyl)-thiazole* (**SA01**): pale gray powder, mp = 230 °C; yield = 85.8%; FT **IR** (KBr) ν_max_ cm^−1^: 1463.71 (C-N), 1571.22 (C=C), 1619.91 (C=N), 1214.45 (C-O, OCH_3_), 3235.97 (N-H); **MS**: *m/z* = 514.3 [M + H]^+^; **^1^H-NMR** (DMSO-*d*_6_, 500 MHz) *δ* ppm: 3.796 (s, 3H, Ar^2^-OCH_3_), 4.000 (s, 3H, Q_6_-OCH_3_), 4.015 (s, 3H, Q_7_-OCH_3_), 6.980 (d, 2H, Ar^2^-H_3_, *J* = 9 Hz), 7.170 (s, 1H, Th-H_5_), 7.400–7.418 (m, 3H – 2H Ar^1^-H_3_, 1H Q-H_8_), 7.610 (s, 1H, Q-H_5_), 7.776–7.807 (m, 4H – 2H Ar^1^-H_2_, 2H Ar^2^-H_2_), 8.096 (s, 1H, -CH=N-), 8.637 (s, 1H, Q-H_2_); **^13^C-NMR** (DMSO-*d*_6_, 125 MHz) *δ* ppm: 55.111 (Ar^2^-OCH_3_), 56.091 (Q_7_-OCH_3_), 56.252 (Q_6_-OCH_3_), 100.824 (Q_8_), 104.631 (Th-C_5_), 106.129 (Q_4a_), 109.741 (Q_5_), 113.954 (Ar^2^-C_3_), 122.199 (Ar^2^-C_1_), 122.640 (Ar^1^-C_3_), 126.833 (Ar^2^-C_2_), 127.484 (Ar^1^-C_2_), 132.033 (Ar^1^-C_1_), 150.295 (Q_6_), 151.995 (Q_7_), 152.962 (Ar^1^-C_4_), 156.076 (Q_4_), 158.785 (Ar^2^-C_4_), 164.874 (Q_2_), 168.052 (Th-C_2_);

*(E)-2-(2-(4-((6,7-dimethoxyquinazolin-4-yl)oxy)benzylidene)hydrazineyl)-4-(4-fluorophenyl)-thiazole* (**SA02**): pale yellow powder, mp = 242–243 °C; yield = 73.7%; FT **IR** (KBr) ν_max_ cm^−1^: 1468.53 (C-N), 1569.29 (C=C), 1617.98 (C=N), 1215.42 (C-O, OCH_3_), 3236.45 (N-H); 1092.96 (C-F); **MS**: *m/z* = 502.2 [M + H]^+^; **^1^H-NMR** (DMSO-*d*_6_, 500 MHz) *δ* ppm: 4.001 (s, 3H, Q_6_-OCH_3_), 4.016 (s, 3H, Q_7_-OCH_3_), 7.251 (t, 2H, Ar^2^-H_3_, *J* = 9 Hz), 7.331 (s, 1H, Th-H_5_), 7.404–7.421 (m, 3H – 2H Ar^1^-H_3_, 1H Q-H_8_), 7.611 (s, 1H, Q-H_5_), 7.785 (d, 2H, Ar^1^-H_2_, *J* = 8.5 Hz), 7.906 (dd, 2H, Ar^2^-H_3_, *J*_1_ = 2.75 Hz, *J*_2_ = 8.75 Hz), 8.108 (s, 1H, -CH=N-), 8.639 (s, 1H, Q-H_2_); **^13^C-NMR** (DMSO-*d*_6_, 125 MHz) *δ* ppm: 56.091 (Q_7_-OCH_3_), 56.259 (Q_6_-OCH_3_), 100.824 (Q_8_), 104.540 (Th-C_5_), 106.118 (Q_4a_), 109.741 (Q_5_), 115.424 (Ar^2^-C_3_, *J* = 21 Hz), 122.647 (Ar^1^-C_3_), 127.442 (Ar^1^-C_1_), 127.519 (Ar^2^-C_2_), 127.736 (Ar^2^-C_1_), 131.997 (Ar^1^-C_2_), 149.441(Q_8a_), 150.302 (Q_6_), 151.981 (Q_7_), 153.003 (Ar^1^-C_4_), 156.083 (Q_4_), 161.517 (Ar^2^-C_4_, *J* = 228 Hz), 164.874 (Q_2_), 168.269 (Th-C_2_).

*(E)-2-(2-(4-((6,7-dimethoxyquinazolin-4-yl)oxy)benzylidene)hydrazineyl)-4-(4-chlorophenyl)-thiazole* (**SA03**): pale yellow powder, mp = 231–232 °C; yield = 96.2%; FT **IR** (KBr) ν_max_ cm^−1^: 1463.22 (C-N), 1564.95 (C=C), 1618.95 (C=N), 1215.9 (C-O, OCH_3_), 3236.45 (N-H); 742.94 (C-Cl); **MS**: *m/z* = 518.2 [M + H]^+^; **^1^H-NMR** (DMSO-*d*_6_, 500 MHz) *δ* ppm: 4.000 (s, 3H, Q_6_-OCH_3_), 4.015 (s, 3H, Q_7_-OCH_3_), 7.405–7.420 (m, 4H – 2H Ar^2^-H_3_, 1H Q-H_8_, 1H Th-H_5_), 7.479 (d, 2H, Ar^1^-H_3_, *J* = 9 Hz), 7.610 (s, 1H, Q-H_5_), 7.878 (d, 2H, Ar^2^-H_2_, *J* = 8.5 Hz), 7.791 (d, 2H, Ar^1^-H_2_, *J* = 9 Hz), 8.111 (s, 1H, -CH=N-), 8.637 (s, 1H, Q-H_2_); **^13^C-NMR** (DMSO-d_6_, 125 MHz) *δ* ppm: 56.091 (Q_7_-OCH_3_), 56.259 (Q_6_-OCH_3_), 100.817 (Q_8_), 104.533 (Th-C_5_), 106.129 (Q_4a_), 109.741 (Q_5_), 122.654 (Ar^1^-C_3_), 127.204 (Ar^1^-C_1_), 127.540 (Ar^2^-C_3_), 128.611 (Ar^2^-C_2_), 128.660 (Ar^2^-C_1_), 131.907 (Ar^2^-C_4_), 131.944 (Ar^1^-C_2_), 149.791 (Q_8a_), 150.295 (Q_6_), 151.988 (Q_7_), 153.024 (Ar^1^-C_4_), 156.076 (Q_4_), 164.867 (Q_2_), 168.311 (Th-C_2_).

*(E)-2-(2-(4-((6,7-dimethoxyquinazolin-4-yl)oxy)benzylidene)hydrazineyl)-4-(4-(trifluoromethyl)-phenyl)thiazole* (**SA04**): pale yellow powder, mp = 237–238 °C; yield = 90%; FT **IR** (KBr) ν_max_ cm^−1^: 1466.12 (C-N), 1569.29 (C=C), 1617.98 (C=N), 1216.86 (C-O, OCH_3_), 3239.48 (N-H); 1070.30 (C-F); **MS**: *m/z* = 552.2 [M + H]^+^; **^1^H-NMR** (DMSO-*d*_6_, 500 MHz) *δ* ppm: 4.003 (s, 3H, Q_6_-OCH_3_), 4.017 (s, 3H, Q_7_-OCH_3_), 7.411–7.428 (m, 3H – 2H Ar^1^-H_3_, 1H Q-H_8_), 7.608 (d, 2H, Ar^2^-H_3_, *J* = 6 Hz), 7.775–7.810 (m, 4H – 1H Q-H_5_, 2H Ar^1^-H_2_, 1H Th-H_5_), 8.085 (d, 2H, Ar^2^-H_2_, *J* = 8 Hz), 8.123 (s, 1H, -CH=N-), 8.648 (s, 1H, Q-H_2_); **^13^C-NMR** (DMSO-*d*_6_, 125 MHz) *δ* ppm: 56.105 (Q_7_-OCH_3_), 56.266 (Q_6_-OCH_3_), 100.838 (Q_8_), 106.024 (Th-C_5_), 106.542 (Q_4a_), 109.741 (Q_5_), 122.119 (CF_3_, *J* = 288 Hz), 122.661 (Ar^1^-C_3_), 125.062 (Ar^2^-C_3_), 126.035 (Ar^2^-C_2_), 127.575 (Ar^1^-C_1_), 129.479 (Ar^2^-C_4_, *J* = 47.25 Hz), 131.921 (Ar^1^-C_2_), 150.323 (Q_6_), 151.953 (Q_7_), 153.045 (Ar^1^-C_4_), 155.453 (Q_4_), 164.902 (Q_2_), 168.479 (Th-C_2_);

*(E)-2-(2-(4-((6,7-dimethoxyquinazolin-4-yl)oxy)benzylidene)hydrazineyl)-4-(4-benzonitrile)thiazole* (**SA05**): pale brown powder, mp = 243–244 °C; yield = 86.7%, FT **IR** (KBr) ν_max_ cm^−1^: 1469.49 (C-N), 1558.64 (C=C), 1618.47 (C=N), 1209.15 (C-O, OCH_3_), 3235.49 (N-H); 2221.59 (C≡N); **MS**: *m/z* = 509.4 [M + H]^+^; **^1^H-NMR** (DMSO-*d*_6_, 500 MHz) *δ* ppm: 4.005 (s, 3H, Q_6_-OCH_3_), 4.020 (s, 3H, Q_7_-OCH_3_), 7.413–7.431 (m, 3H – 2H Ar^1^-H_3_, 1H Q-H_8_), 7.620 (s, 1H, Q-H_5_), 7.670 (s, 1H, Th-H_5_), 7.800 (d, 2H, Ar^2^-H_3_, *J* = 8.5 Hz), 7.883 (d, 2H, Ar^1^-H_2_, *J* = 9 Hz), 8.049 (d, 2H, Ar^2^-H_2_, *J* = 8.5 Hz), 8.127 (s, 1H, -CH=N-), 8.671 (s, 1H, Q-H_2_); **^13^C-NMR** (DMSO-*d*_6_, 125 MHz) *δ* ppm: 56.133 (Q_7_-OCH_3_), 56.301 (Q_6_-OCH_3_), 100.887 (Q_8_), 105.758 (Th-C_5_), 107.613 (Q_4a_), 109.580 (Q_5_), 109.741 (Ar^2^-C_4_), 118.966 (-CN), 122.647 (Ar^1^-C_3_), 126.098 (Ar^2^-C_2_), 131.935 (Ar^1^-C_2_), 132.684 (Ar^2^-C_3_), 138.718 (Ar^2^-C_1_), 148.832 (Q_8a_), 150.386 (Q_6_), 151.862 (Q_7_), 153.031 (Ar^1^-C_4_), 156.027 (Q_4_), 164.993 (Q_2_), 168.500 (Th-C_2_).

*(E)-2-(2-(4-((6,7-dimethoxyquinazolin-4-yl)-oxy)-benzylidene-)hydrazineyl)-4-methyl-thiazole* (**SA06**): pale yellow powder, mp = 253–252 °C; yield = 86%; FT **IR** (KBr) ν_max_ cm^−1^: 1457.92 (C-N), 1616.06 (C=N), 1211.56 (C-O, -OCH_3_), 3232.59 (N-H); 3079.76 (C-H_,_ -CH_3_); **MS**: *m/z* = 422.3 [M + H]^+^; **^1^H-NMR** (DMSO-*d*_6_, 500 MHz) *δ* ppm: 2.178 (s, 1H, -CH_3_), 3.987 (s, 3H, Q_6_-OCH_3_), 4.001 (s, 3H, Q_7_-OCH_3_), 6.387 (br. S, 1H, Th-H_5_), 7.371–7.402 (m, 3H – 2H Ar^1^-H_3_, 1H Q-H_8_), 7.576 (s, 1H, Q-H_5_), 7.747 (d, 2H, Ar^1^-H_2_, *J* = 8.5 Hz), 8.059 (s, 1H, -CH=N-), 8.568 (s, 1H, Q-H_2_); **^13^C-NMR** (DMSO-*d*_6_, 125 MHz) *δ* ppm: 14.705 (Th-CH_3_), 56.007 (Q_7_-OCH_3_), 56.161 (Q_6_-OCH_3_), 100.670 (Q_8_), 104.316 (Th-C_5_), 106.745 (Q_4a_), 109.727 (Q_5_), 122.612 (Ar^1^-C_3_), 127.386 (Ar^1^-C_2_), 132.117 (Ar^1^-C_1_), 148.923 (Q_8a_), 150.127 (Q_6_), 152.177 (Q_7_), 152.993 (Ar^1^-C_4_), 155.803 (Q_4_), 164.643 (Q_2_), 168.542 (Th-C_2_).

*(E)-5-(2-(2-(4-((6,7-dimethoxyquinazolin-4-yl)oxy)benzylidene)hydrazineyl)thiazol-4-yl)-2-hydroxy-benzamide* (**SA07**): pale yellow powder, mp = 246–247 °C; yield = 77.8%; FT **IR** (KBr) ν_max_ cm^−1^: 1472.87 (C-N), 1618.47 (C=N), 1221.2 (C-O, -OCH_3_), 3236.45 (N-H), 1672.46 (C=O); **MS**: *m/z* = 543.3 [M + H]^+^; **^1^H-NMR** (DMSO-*d*_6_, 500 MHz) *δ* ppm: 4.001 (s, 3H, Q_6_-OCH_3_), 4.016 (s, 3H, Q_7_-OCH_3_), 6.939 (d, 1H, Ar^2^-H_3_, *J* = 8.5 Hz), 7.173 (s, 1H, Th-H_5_), 7.405–7.422 (m, 3H – 2H Ar^1^-H_3_, 1H Q-H_8_), 7.612 (s, 1H, Q-H_5_), 7.790 (d, 2H, Ar^1^-H_2_, *J* = 8.5 Hz), 7.895 (dd, 1H, Ar^2^-H_6_, *J*_1_= 2 Hz, *J*_2_= 9 Hz), 7.948 (br. S, 1H, -CONH_2_), 8.113 (s, 1H, -CH=N-), 8.348 (d, 1H, Ar^2^-H_4_, *J* = 2 Hz), 8.482 (br. S, 1H, -CONH_2_), 8.643 (s, 1H, Q-H_2_); **^13^C-NMR** (DMSO-*d*_6_, 125 MHz) *δ* ppm: 56.098 (Q_7_-OCH_3_), 56.259 (Q_6_-OCH_3_), 100.824 (Q_8_), 104.057 (Th-C_5_), 106.080 (Q_4a_), 109.741 (Q_5_), 114.661 (Ar^2^-C_3_), 117.443 (Ar^2^-C_1_), 122.640 (Ar^1^-C_3_), 125.433 (Ar^2^-C_5_), 127.491 (Ar^1^-C_2_), 131.299 (Ar^1^-C_1_), 131.285 (Ar^2^-C_6_), 132.026 (Ar^2^-C_4_), 150.309 (Q_6_), 151.974 (Q_7_), 152.975 (Ar^1^-C_4_), 156.097 (Q_4_), 160.213 (Ar^2^-C_2_), 164.888 (Q_2_), 168.213 (Th-C_2_), 171.733 (CONH_2_).

### 3.3. In Vitro Cytotoxicity Evaluation

A human endothelial hybrid cell line (EA.hy926) (passage number 10–15), human hepatocellular carcinoma (HepG2) cells (passage number 10–15), and normal foreskin fibroblasts (BJ) (passage number 5–10) were used in the present study. All cell lines were purchased from ATCC (Manassas, United States of America). EA.hy926 cells were maintained in DMEM with high glucose supplemented with 1% sodium pyruvate, while the BJ cells were maintained in DMEM with low glucose concentration. HepG2 cells were cultured in MEM. All media were supplemented with 10% fetal bovine serum (FBS) and 1% penicillin/streptomycin. All cell lines were cultured at 37 °C in an incubator with 5% CO_2_ supplementation and cellular media were refreshed every other day. Once the cells reached a confluency of 80–90%, they were either subcultured or used in experiments.

The synthesized compounds’ cytotoxicity was evaluated using the Alamar Blue (AB) assay as previously described [37]. The metabolically active cell-dependent conversion of non-fluorescent resazurin to the highly fluorescent resorufin compound is quantified spectrophotometrically at *λ_excitation_ =* 530/25; *λ_emission_ =* 590/35, using a Synergy 2 Multi-Mode microplate reader. Briefly, a total of 5 x 10^3^ EA.hy926 and BJ cells and 1 x 10^4^ HepG2 cells were seeded in 100 µL in 96 well plates. The cells were left to attach overnight. The attached cells were washed with PBS and further exposed to **sorafenib** and the synthesized compounds, namely **SA01–SA07,** at different ranges of concentrations. At 48 h post-stimulation, the media were removed, and the AB assay was performed. The reported range of concentrations for each compound was obtained by employing the up-and-down method. 

The experiments were conducted in three biological replicates, each one including 6 technical replicates. Cells exposed to culture media with 0.2% DMSO served as negative control (NC) and were used for data normalization (100%). The 50% Inhibitory Concentration (IC_50_) values were determined based on the dose–response curves obtained by fitting the experimental data with a 4-parameter logistic curve in SigmaPlot 11 software.

### 3.4. Molecular Docking Studies

The crystal structure of human VEGFR2 co-crystallized with **sorafenib** was taken from the Protein Data Bank (PDB code 4ASD obtained by X-ray diffraction with 2.03 Å resolution) [26,29]. The deposited protein macromolecule from the respective complex comprised the juxtamembrane domain and the catalytic tyrosine kinase domains (the ATP-binding domain, the kinase insert domain, and the phosphotransferase domain). Because some amino acids were missing from the crystalized structure, 3D homology modeling of the target protein was employed with the SWISS-MODEL using the respective structure as a template (GMQE = 0.89) [38]. The resulting protein was used as a target in the molecular docking study performed using AutoDock Vina 1.1.2 after preparation by a method previously reported [39,40,41]. 

The search space was defined as a cube with the coordinates of the center x = −21.966, y = −0.473, and z = −11.442 and sides equal to 20, according to an adaptation of a previously reported protocol [42]. The parameters’ exhaustiveness and num_modes were set to 50 and 20, respectively. The parameters of the search space were chosen to include the previously co-crystallized **sorafenib** from the template structure and the amino acids important for ATP-binding from the kinase domain indicated by blastp [43]. 

The files containing the 3D structures of the compounds **SA01–SA07** and **sorafenib** used as reference compound were obtained after energy minimization in Section 3.7 from the DFT calculations. The dataset of three-dimensional structures of ligands were processed using AutoDock Tools 1.5.6 [41] by addition of the Gasteiger charges and removal of the non-polar hydrogen atoms, as previously reported [40,41,44].

The top binding pose of **sorafenib** in the active site was in superposition with the initial co-crystallized **sorafenib** molecule after visual inspection and was confirmed after the computation of the RMSD of the coordinates of the heavy atoms, which was equal to 1.22 Å [45]. The visualization of the results of the molecular docking study was performed using UCFS Chimera 1.10.2 [46].

### 3.5. Molecular Dynamics Studies 

To get a better insight into the interactions between the ligands, the evolution in time of the predicted complexes compounds with VEGFR2 was studied in a molecular dynamics study using GROMACS 2023 for 100 ns using a CHARMM36 force field on a machine running Debian 11 with CUDA 12 for operating an NVIDIA RTX 3060 GPU [47,48,49,50,51]. For each constructed complex, the top binding conformation of each ligand and their parametrization was performed using CgenFF [47]. The systems were electrically neutralized after solvation using the TIP3P water model in an orthorhombic box with a 1 nm gap. To avoid collisions among atoms, using the steepest descent method, the system’s energy was minimized (converge criterion <1000 KJ mol^−1^ nm^−1^). Equilibration of the systems was performed at NVT and NPT ensembles at 300 K for 100 ps, according to the previous works reported and the simulations were run with periodic boundary conditions on all axes [52,53,54]. During the simulation a velocity-rescale thermostat [55] and Parrinello–Rahman barostat [56] were used for temperature and pressure coupling, with 300K reference temperature and 1 bar reference pressure, respectively [57,58].

Visual analysis of the trajectories was performed using VMD 1.9.4 [39]. Regarding the numerical evaluation of the stability of the predicted complexes, trajectories were evaluated using the built-in functions of GROMACS and VMD 1.9.4.

### 3.6. MM-PBSA Free Energy Calculation 

The average free binding energy calculation of the ligands **SA02–SA05** and **sorafenib** to the VEGFR2 was computed based on the Molecular Mechanics–Poisson–Boltzmann Surface Area (MM-PBSA) using gmx_MMPBSA [59]. The last 25 ns of each MD simulation (all the last 2500 frames with no intervals) were analyzed to assess the energy involvement of each amino acid found within 5 Å using the energy decomposition strategy [60]. The energy variation between the ligand to protein and ligand unbound to the protein was computed using the formula ΔG_binding_ = G_complex_ – (G_complex_ + G_ligand_) [49,61]. 

### 3.7. Density Function Theory (DFT) Calculations 

The DFT in silico calculations were performed with the B3LYP functional and the 6-311+G* basis set using Spartan 20 (Wavefunction, Inc., Irvine, CA, USA) to identify the electronic and structural characteristics of the compounds **SA01–SA07** and **sorafenib** used as reference drug [61]. The calculations were performed to identify the regions of the molecules important for their interaction with VEGFR2 according to their electron density, angles, frontier molecular orbitals (FMOs), and dipole momentum. The global reactivity descriptors (GRDs) (I, A, μ, S, η, ω, N, ∆N) were also calculated [62]. 

### 3.8. Wound Healing (Scratch) Assay

EA.hy926 cells were seeded in 12-well plates and incubated for 24 h until confluency was reached. The medium was removed and a scratch in the cell monolayer was manually added with a sterile pipette tip. Subsequently, the cells were gently washed two times with DPBS to remove the detached cells. The cell monolayer was further exposed to **SA04** (0.25, 0.5, 0.75, 1 μM), **SA05** (0.75, 1, 1.25, 1.5 μM), **sorafenib** (1.25, 2.5, 5 μM), or the control (0.2% DMSO). Images were captured at the time of exposure (T0) and 12 h post-exposure (T12). The rate of cell migration was determined by calculating the wound healing percentage (%) according to the formula (A_T0_ – A_T12_)/ A_T0_*100 previously reported [63]. The scratch area at the initial time of exposure (A_T0_) and after 12 h (A_T12_), expressed as square pixels (Px^2), were determined using the IKOSA Prisma Application Wound Healing (Scratch) assay (v2.2.0) [64].

### 3.9. Chorioallantoic Egg Membrane (CAM) Assay 

To assess the anti-angiogenic effects, a biological model represented by the chorioallantoic membrane (CAM) of chicken eggs was employed. The CAM was exposed to the test substances: **sorafenib, SA04**, **SA05** (1.5 μM), and the control sample (0.2% DMSO). The experimental procedure involved the following steps: (I) initial cleaning with 70% alcohol solution and incubation of the eggs at a constant humidity (45%) and temperature (37.5 °C) on the first day; (II) on the third day of embryonic development (EDD3), a small hole at the egg tip was created and approximately 5–7 mL of albumen were extracted to facilitate detachment of the chorioallantoic membrane from the inner shell; and (III) on EDD4, a window was created at the egg’s top to visualize the vascular plexus, covering it with adhesive tape, and returning the egg to the incubator until the experiment’s commencement.

The assessment of anti-angiogenic impact began on the sixth day of embryonic development and extended over five days. To achieve this, a silicon ring was applied to the CAM, and 10 μL of the tested compounds was administered daily within the ring. The evaluation of angiogenesis involved daily photographic documentation of the vascular plexus using a Discovery v.8 stereomicroscope, a Zeiss Axio CAM 105 color camera, and ZEN core 3.8 software. For the quantitative assessment of the anti-angiogenic potential of **SA04, SA05**, and **sorafenib** compared to the control sample, a total area of 8*10^6^ square pixels (Px^2^) around the area isolated by the silicon ring was selected. The quantitative evaluation of vessel numbers branching points and total vessel area was performed using the IKOSA Prism Application CAM assay (v3.1.0) [65].

### 3.10. In Silico Physicochemical and Pharmacokinetics Predictions

The physicochemical, drug-likeness, and ADME (absorption, distribution, metabolism, elimination) profiles of the synthesized compounds **SA01-SA07** and **sorafenib** were evaluated using the SwissADME web tool [66].

## 4. Results and Discussion 

### 4.1. Chemistry

**Figure 3:**
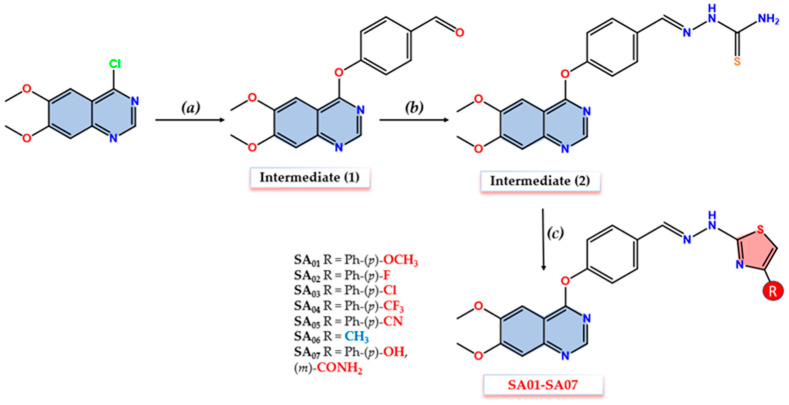
Synthetic procedure for the quinazoline–thiazole hybrid compounds SA01-SA07. (a) The 4-hydroxy-benzaldehyde, K_2_CO_3_, MeCN, reflux 10 h; (b) thiosemicarbazide, ethanol, H_2_SO_4_ conc., reflux 20 h; (c) corresponding α-haloketones, acetone/DMF 10:1, rt 8 h or reflux.

Seven novel quinazoline–thiazole (**SA01–SA07**) hybrid derivatives were synthesized according to the synthetic route depicted in Figure 3. The aldehyde intermediate (**1**) was synthesized in a good yield through the oxygen-alkylation of 4-hydroxy-benzaldehyde with 6,7-dimethoxy-4-chloro-quinazoline using a modified protocol previously reported [67]. Acetonitrile (MeCN) was used as a solvent, and the alkaline pH was provided by an excess (1:3 ratio) of potassium carbonate (K_2_CO_3_). The thiosemicarbazone intermediate (**2**) was synthesized according to a modified protocol reported by Marc et al. [35]. A ratio of 2:1 thiosemicarbazide to aldehyde derivative (**1**) was necessary to synthesize intermediate (**2**) in a satisfying yield with corresponding purity, due to limited progress of the reaction with a 1:1 ratio after 48 h with H_2_SO_4_ conc. used as a catalyst. The excess use of thiosemicarbazide was a favorable compromise due to its increased water solubility which made it easy to remove in the final phase of the isolation through repeated water rinsing. 

The final compounds **SA01-SA07** were synthesized via cyclization to the thiazole ring of the thiosemicarbazone moiety of intermediate (**2**) and various α-haloketones as previously reported [35]. The synthetic procedure was adopted using acetone and dimethylformamide (DMF) (10:1 ratio) to address the low solubility of intermediate (**2**). The synthesis of compounds **SA01**, **SA03–SA05,** and **SA07** in a good yield was achieved by magnetic stirring, at room temperature (rt) for 8 h, of intermediate (**2**) in the presence of the corresponding α-haloketones after the prior solubilization in the solvent mixture by stirring for 1 h. For compound **SA02,** a similar protocol was followed, with additional reflux for two more hours, due to the limited progress of the reaction as monitored by TLC, to achieve a satisfying yield. For **SA06**, the synthesis was achieved under reflux conditions with the dropwise addition of chloroacetone to the reaction flask after the prior solubilization of intermediate (**2**).

All the intermediates (**1,2**) and the final compounds (**SA01–SA07**) were assessed by spectral data (Supplementary Figures S1–S36), and they are consistent with the proposed structures. In the MS spectra of all the compounds, the molecular peak was identified as [M + H]^+^. The formation of intermediate (**1**) through O-alkylation was confirmed in the FT-IR spectra by the appearance of a high-intensity signal corresponding to the C=O bond at 1702.28 cm^−1^ followed by the disappearance of the same peak in the FT-IR spectra of intermediate (**2**) confirming the blockage of the carbonyl group. In addition to the hydrazine N-H signal consistent in intermediate (**2**) and **SA01–SA07** (3232.59–3251.59 cm^−1^), specific signals were also identified that confirm the final synthesis step completion: C-F bond signals at 1092.96 cm^−1^ (**SA02**) and 1070.30 cm^−1^ (**SA04**), a C-Cl bond signal at 1092.96 cm^−1^ (**SA03**), a C≡N bond signal at 2221.59 cm^−1^ (**SA05**), an aliphatic C-H bond signal at 3079.76 cm^−1^ (**SA06**), and an amide C=O at 1672.46 cm^−1^ (**SA07**). Corresponding signals related to quinazoline core such as C-N (1461.78–1474.8 cm^−1^), C=C (1558.64–1578.45 cm^−1^), and C=N (1617.5–1619.91 cm^−1^) remained consistent in all intermediates and final compounds. 

In the hydrogen nuclear magnetic resonance (^1^H-NMR) spectra, all the desired peaks for the corresponding compounds were found with expected coupling and multiplicity. The identified peaks for intermediate (**1**) are according to a previous report [67]. The presence of quinazoline-H_2_ as a singlet peak (8.571–8.671 ppm) and the two methoxy groups in positions 6 and 7 as two singlets (3.796–4.005, 4.000–4.020 ppm) were consistent among all the synthesized compounds. A distinctive peak corresponding to thiazole-H5 proton appears in all the final compounds’ ^1^H-NMR spectra, confirming the ring closure along with specific peaks for **SA03** (3.796 ppm, singlet, 4-methoxy), **SA06** (2.178 ppm, methyl), and **SA07** (two broad singlets at 7.948 and 8.482 ppm, -CONH_2_). The multicentric electronic conjugation effect of the various substituents on the distal aromatic moiety (**SA01–SA05, SA07**) is identified not only on the respective nucleus but also at a distance. Significant deshielding effects were present on the two protons in *meta* relative to the EWG-substituted derivatives (**SA04**, 8.085 ppm / **SA05**, 8.049 ppm) compared to the EDG-substituted derivative (**SA01**, 6.980 ppm). The same phenomenon can be identified for thiazole-H5 and azomethine protons, diminished due to the distance. The azomethine proton (-CH=N-) was identified as a singlet in intermediate (**2**) and **SA01–SA07** (8.059–8.133 ppm) confirming the synthesized compounds are found as *E*-isomers according to the literature [68,69]. In the ^13^C-NMR spectra, the thiazole ring formation was confirmed by the appearance of two distinctive peaks corresponding to thiazole-C5 (104.057–106.026 ppm) and C2 (168.052–168.542 ppm).

### 4.2. In Vitro Cytotoxicity Evaluation

**Figure 4:**
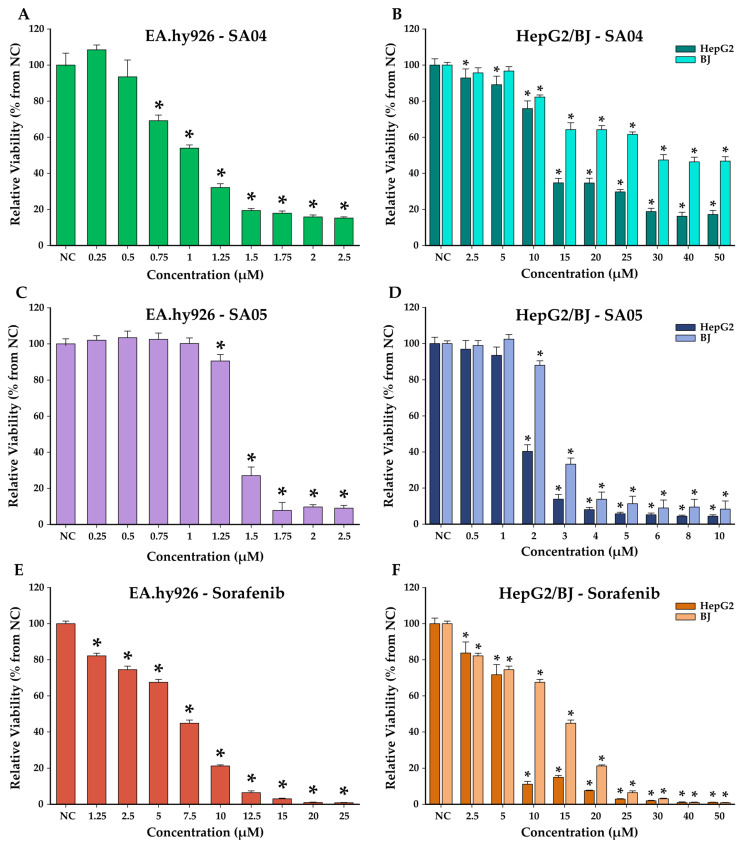
Cytotoxic effect of **SA04** (**A**,**B**), **SA05** (**C**,**D**), and **sorafenib** (**E**,**F**) after a 48 h exposure of EA.hy926, HepG2, and BJ cells. The results are expressed as relative means ± standard deviations of three biological replicates. Data are expressed as relative values compared to the negative control (NC) (100%). Asterisks (*) indicate significant differences (*p* < 0.05) compared to NC.

The cytotoxic effects of the compounds **SA01–SA07** were evaluated on human hepatocarcinoma cells (HepG2), immortalized endothelial cells (EA.hy926), and normal human foreskin cells (BJ) after a 48 h exposure using the VEGFR2 inhibitor **sorafenib** as a reference drug. The dose-dependent decrease in cellular viability for the most promising compounds **SA04** and **SA05** along with the reference drug **sorafenib** on the three cell lines is depicted in Figure 4 (for compounds **SA01–SA03** and **SA06–SA07** see Supplementary Figures S36–S37). 

HepG2 cells’ dependency on VEGFR2 signaling for proliferation and migration, along with the upregulation of VEGFR2, makes them an ideal in vitro model for the preliminary evaluation of series **SA01–SA07** [70,71]. **Sorafenib** provides a suitable reference drug due to its widespread clinical use in the treatment of advanced forms of hepatocellular carcinoma [72]. 

Four of the seven evaluated compounds (**SA02–SA05**) displayed superior antiproliferative activity (IC_50_ =1.83–4.24 µM) compared to the reference drug **sorafenib** (IC_50_ = 6.28 µM) towards HepG2 cells, with the most active compound of the series (**SA05**) displaying a 3.5-fold higher activity compared to **sorafenib**. The provided data suggest that the 4-phenyl substitution of the thiazole ring is beneficial for the antiproliferative activity towards HepG2 cells.

The 4-Methyl-thiazole derivative **SA06** displayed a low cytotoxic potential towards the entire cell panel (Table 1). Aside from the inferior cytotoxic activity of **SA06**, the limited solubility in the cellular media observed with concentrations higher than 5 µM, could explain the lack of proportionality between the dose and the measured cytotoxicity in all three cell types. The substitution pattern on the 4-phenyl-thiazole moiety seems to play a critical role in the antiproliferative activity. Compounds **SA02–SA05** with electron-withdrawing groups (EWGs) in *para* (-F, -Cl, CF_3_, -CN) displayed significantly higher antiproliferative activity compared to **sorafenib**, while the presence of the electron-donor groups (EDGs) in **SA01** (*para* -OCH_3_) and **SA07** (3-amido-4-hydroxy) had a negative effect on their activities. 

The proliferation, migration, and differentiation of the vascular endothelial cells are fundamental processes for tumor angiogenesis [6,12]. To assess the proliferation inhibition potential of the synthesized compounds **SA01–SA07** and **sorafenib**, an in vitro model to assess the IC_50_ was employed using the EA.hy926 cell line (Table 1). These cells were obtained by the fusion of HUVEC (human umbilical vein endothelial cells) and the cancer cells A549/8, providing the phenotype of the endothelial cells and the immortality of the tumor cells. EA.hy926 retain most of the features of HUVEC such as endothelial adhesion molecule expression, human factor VIII-related Ag, and VEGFR2 signaling, providing a suitable model to assess the anti-angiogenic potential of the series **SA01–SA07** [73,74]. All the 4-phenyl-thiazole derivatives (**SA01–SA05**, **SA07**) displayed superior activity (IC_50_ = 0.79–5.85 µM) compared to the reference drug **sorafenib** (IC_50_ = 6.62 µM). A similar structure–activity relationship pattern to HepG2 cells can be identified in the case of EA.hy296, with superior activity for the *para*-EWG substitution on the 4-phenyl-thiazole moiety of up to 8.3-fold higher activity compared to **sorafenib** for the most active compound **SA03**. 

Skin toxicity is a widely associated side effect in clinical practice with VEGFR2 inhibitory therapy in various types of tumors [75]. The toxic potential towards BJ cells of **SA01–SA07** and **sorafenib** is depicted in Table 1. While most of the compounds (except **SA06–SA07**) displayed a lower IC_50_ value (2.35–6.79 µM) compared to **sorafenib**, which may involve a higher toxic potential, it is important to outline the selectivity ratio towards the other cells (HepG2, EA.hy296). **SA04** proves itself with a superior index selectivity (SI_a_, SI_b_) compared to that of reference drug sorafenib while having consistently higher antiproliferative activity on both cell lines. 

### 4.3. Molecular Docking Studies

**Figure 5:**
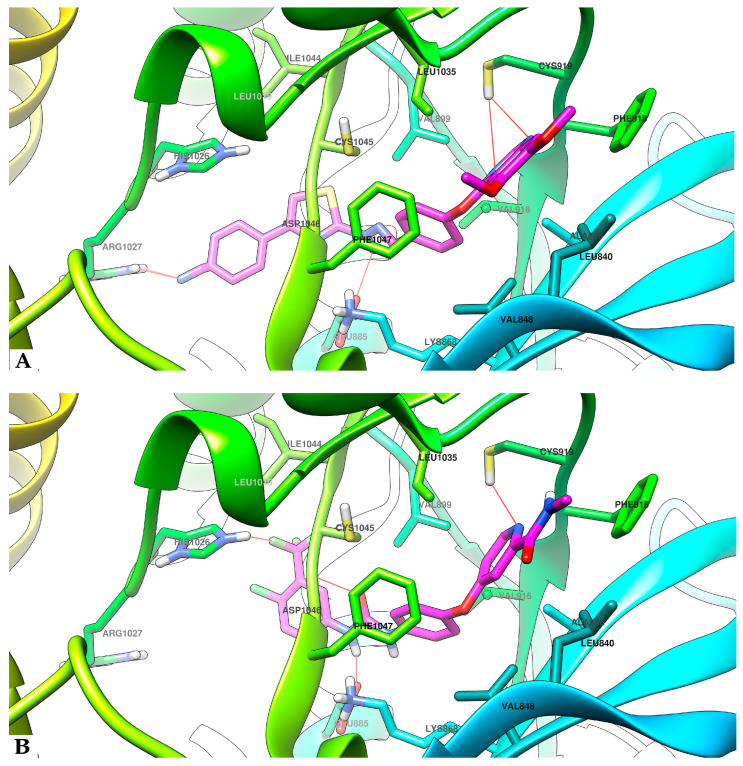
Visualization of the binding mode of (**A**) **SA05** and (**B**) **sorafenib** in the active site of human VEGFR2 kinase domain co-crystalized in the complex 4ASD from PDB using UCFS Chimera 1.10.2. The molecular skeleton is colored with magenta and H-bonds are indicated by thick red lines.

The studied compounds **SA01–SA07** and **sorafenib** as reference compound were docked into the ATP-binding pocket from the VEGFR2. Visual analysis of the top binding poses of the compounds indicated a high degree of superposition between them, sharing some binding features with **sorafenib,** which will be further discussed for comparison. The binding poses of the most promising compound (**SA05**) and **sorafenib** in the active site of VEGFR2 and the superposition of **SA05** with **sorafenib** are indicated in Figure 5.

First, the quinazoline core from **SA01–SA07** has a similarity in terms of binding interaction with the picolinamide fragment of **sorafenib** in the hinge region. The quinazoline moiety of all the evaluated compounds interacts with the Cys919 sidechain via an H-bond to the quinazoline nitrogen atoms in a similar manner to **sorafenib**. The two methoxy groups in positions 6 and 7 of the quinazoline ring of the compounds **SA01–SA07** extend into the solvent-accessible region.

The 4-aryloxy moieties in all, **SA01–SA07** and **sorafenib,** provide a suitable angle to accommodate them in the hydrophobic pocket and, together with the quinazoline core, are harbored in a mainly hydrophobic region of the ATP-binding site comprising Phe918, Val916, Ala866, Leu840, Val868, Phe1047, Val899, and Leu1035. The 4-aryloxy-quinazoline moieties of **SA01–SA07** and the 4-aryloxy-picolinamide moiety of **sorafenib** are superposable, indicating a common binding pattern between the studied compounds and **sorafenib** and suggesting the successful application of the scaffold hopping approach in the development of the novel series. 

**Figure 6:**
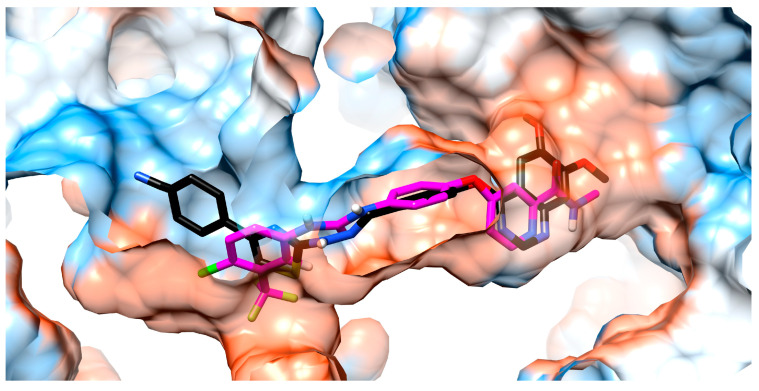
A 3D-surface visualization of the predicted binding mode of **SA05** (black skeleton) superposed with **sorafenib** (magenta skeleton) in the active site of the human VEGFR2 kinase domain co-crystalized in the complex 4ASD from PDB using UCFS Chimera 1.10.2.

The hydrazone linker from the studied compounds **SA01–SA07** is predicted to interact with the sidechain of **Glu885** in the DFG-motif region of the inactive form of the VEGFR2 receptor, involved in the well-characterized salt bridge with the positively charged Lys868 sidechain, crucial for the inhibitory activity [29]. The hydrazone fragment from **SA01–SA07** and the urea fragment of **sorafenib** share a common type of interaction with the backbone of the protein via NH from **Asp1046** as a donor of a H-bond to the hydrazone nitrogen atom as an acceptor. As the superposition of **SA05** and **sorafenib** in the active site of VEGFR2 suggests (Figure 6), the hydrazone moiety occupies the same region as the urea group of **sorafenib** in the gatekeeper region, fulfilling the major criteria of generating both donor and acceptor H-bonds, which is fundamental for VEGFR2 inhibition.

The terminal thiazole substituted in position 4 of **SA01–SA07** corresponds to the 4-chloro-3-trifluoro benzene fragment in **sorafenib**. In the compounds **SA01–SA07,** the mode of various modes of substitution of the thiazole was exploited to identify the importance of substitution in the interaction with the VEGFR2. 

The affinity of the 4-phenyl-thiazole-substituted compounds was higher than that of compound **SA06**, exhibiting just ΔG = 9.8 kcal/mol, the lowest in our present series. This observation leads us to the conclusion that the presence of the benzene ring could be favorable for the affinity of the compounds to VEGFR2. On the other hand, the substitution of the benzene ring with EWGs (**SA02–SA05**) would induce a higher affinity for VEGFR2 (ΔG = 10.3–10.5 kcal/mol) than **SA01**, substituted with an EDG methoxy group (ΔG = 10.1 kcal/mol). Interestingly, the compound substituted with both types of groups (EWG and EDG), **SA07,** is predicted to have the highest theoretical affinity for VEGFR2 (ΔG = 11.0 kcal/mol).

In terms of length, the 4-phenyl-thiazole (**SA01–SA05, SA07**) is bulkier than the chloro-trifluoro benzene fragment from **sorafenib** and according to Figure 6 it dives deeper into the allosteric pocket. Due to the elongation of the molecules, compounds **SA01–SA05** and **SA07** are predicted to have a different way of interacting with VEGFR2 in this region, compared to **sorafenib**, which could be an encouraging fact, because a new binding way could avoid the acquired resistance of cancer cells with mutated VEGFR2 against **sorafenib**. While the trifluoromethyl fragment from **sorafenib** interacts with the positively charged sidechain of His1026, the negatively charged substituents fluorine (**SA02**), chlorine (**SA03**), trifluoromethyl (**SA04**), and nitrile (**SA05**) are predicted to interact with the positively charged sidechain of Arg1027, forming an additional H-bond, in addition to the three bonds already established as key factors for VEGFR2 inhibition. 

### 4.4. Molecular Dynamics Studies 

The molecular dynamics (MD) simulation was performed for the apo VEGFR2 and its complexes with **sorafenib** and **SA01–SA07**. This in silico technique was used to determine the motions of the molecules in a sampled system during simulation, in conditions as close as possible to reality [61]. As the computing power of computers increased in recent years, this technique began to be routinely used to validate molecular docking results to evaluate the degree of stability of the ligand in the target protein [50].

**Figure 7:**
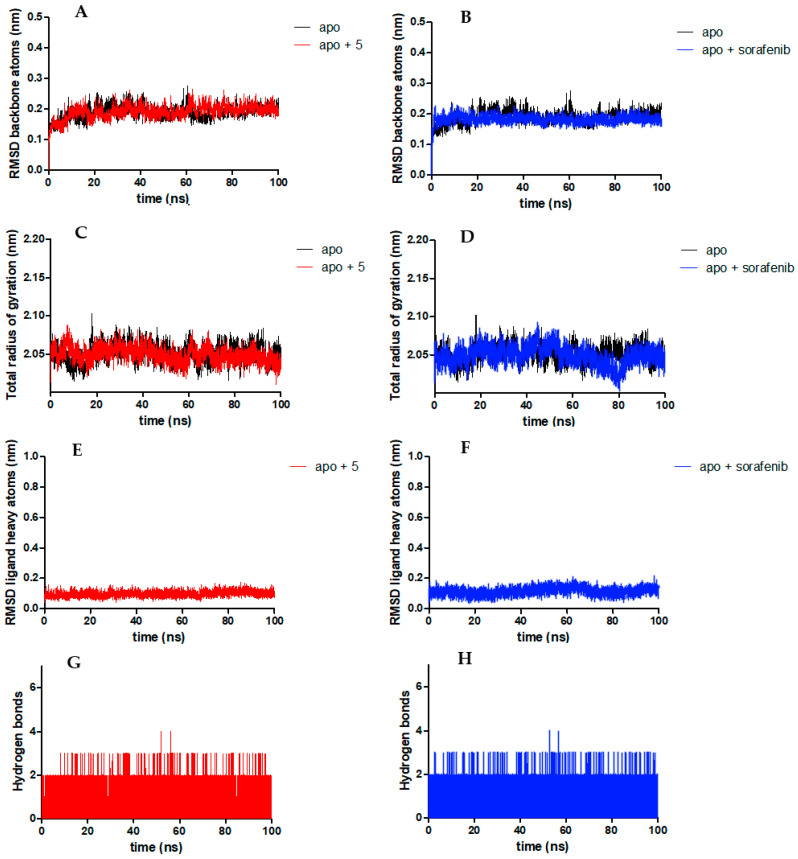
Graphical representation of the MD parameters during the 100 nm simulations: RMSD trajectories of the heavy atoms of VEGFR2 for **SA05** (**A**) and **sorafenib** (**B**); radius of gyration trajectories for **SA05** (**C**) and **sorafenib** (**D**); RMSDs of the ligands’ heavy atoms for **SA05** (**E**) and **sorafenib** (**F**); number of H-bonds encountered for **SA05** (**G**) and **sorafenib** (**H**).

After performing the simulation for 100 ns, the trajectories of the simulations were analyzed, considering the root mean square deviation (RMSD) of the heavy atoms of the VEGFR2, root mean square fluctuation (RMSF) of the amino acids of the protein, RMSD of the ligand, and the radius of gyration (Table 2). A graphical representation of the MD parameters for **SA05** and **sorafenib** is depicted in Figure 7 (for **SA01-SA04** and **SA06-SA07** see Supplementary Figures S39–S42).

In terms of the RMSD of the backbone of the protein, by far the most unstable complex is the one with **SA06**, having the highest RMSD (0.31 nm). All other complexes had RMSDs of the backbone of the protein between 0.16 nm and 0.23 nm, approximately around the RMSD of the apoprotein (0.19 nm), and the corresponding complex with **sorafenib** (0.18 nm). Analysis of the plots of the RMSD of the backbone of the protein in complex with **SA06** indicates a high instability of the complex after 20 ns of simulation, while for the complex with **SA07** after 50 ns and 90 ns of simulation significant changes appear in the backbone of the protein. The complex of **SA05** shares a similar movement with the apoprotein, while **SA03** induces a better stabilization of the complex. 

Analyzing the RMSF of the αCarbon atoms and amino acid sidechain atoms, the highest degree of instability in the current series of compound complexes was identified for the complex of **SA07** with VEGFR2, this having the highest value in our series. For all other compounds complexed, the already mentioned parameters were like the apoprotein or the respective complex with **sorafenib**. Anyway, changes in RMSF should be analyzed carefully because taken out of the context they could give a distorted view regarding the stability of the complexes. Changes in RMSF do not necessarily represent a negative aspect that indicates the instability of the complex, because these changes could be significant conformational changes in the protein as a result of the binding of the inhibitor to it and the severe change in the protein’s structure, leading to its inactivation [76]. 

In terms of the RMSDs of ligands, when docked in complex with VEGFR2, compounds **SA01, SA06,** and **SA07** exhibit the highest movement inside the ATP-binding pocket, with higher RMSDs of the heavy atoms of the ligands (0.12 nm to 0.19 nm) than that of **sorafenib** (0.12 nm) and the other compounds reported in the present paper. The radius of gyration did not add important analytical data about the stability of the predicted complexes with VEGFR2 and does not need to be discussed. For the complex with **SA06**, a repeated significant change was identified during the simulation, indicating a lack of stability.

Compound **SA05** and **sorafenib** bound to the VEGFR2 exhibit impressive stability over time, keeping their position during the simulation, indicating very good stability of the complexes. In the case of **SA01**, some fluctuations of the ligand could be identified which must be interpreted carefully, because it crossed the 0.2 nm maximum tolerable RMSD for the ligand. After 80 ns of simulation, the **SA01** tends to increase its movement, indicating instability of the predicted complex, with a tendency to increase the drift in the 80 ns–100 ns interval of simulation. A constant trend to drift was identified for **SA06** when docked to VEGFR2, because it did not reach the stability of the predicted complex, not even at the end of simulations. 

All compounds interact with amino acid residues of VEGFR2 with approximately one H-bond all along the simulation, except **SA07**, but this did not correlate with other previously mentioned results from the analysis of the MD simulations. Even though **SA07** forms more H-bonds with the protein (1.60 H-bonds/ns), this interaction is not beneficial for the stability of the complex because the parameters we mentioned earlier indicate a significant degree of instability brought to the complex by binding **SA07** into the ATP-binding site. An in-depth analysis of the H-bonds with apo-VEGFR2 during the 100 ns simulation for **SA01–SA07** and **sorafenib** is presented in Table 3. The provided data suggest a different interaction pattern between **sorafenib** and the novel series **SA01–SA07**. While the three major H-bond (Glu885, Cys919, and Asp1046) interactions proven to be vital for VEGFR2 inhibition are present in both the **sorafenib** complex and the **SA01–SA07** complexes, there is a remarkable difference in the distribution. In the hinge region, for the series **SA01–SA07,** the H-bond with Cys919 was 7 to 10 times more frequently encountered during the simulation compared to **sorafenib**. The engagement of the hydrazone moiety of **SA01–SA07** in H-bonds with Glu885 was 2-fold more frequent compared to **sorafenib**, while the Asp1046 interaction was 5-fold more frequent for the urea group of **sorafenib**. In the case of **SA07**, an increased number of interactions with Ile1025 and Arg1027 was identified, which accounts for 35% of the total encountered H-bond interactions.

### 4.5. MM-PBSA Free Energy Calculation 

To provide detailed information about the molecular binding mechanism of compounds that gave stable complexes over time with VEGFR2 during the MD simulation (**SA02-SA05** and **sorafenib**), the binding free energies were calculated by MM-PBSA by processing the last 2500 frames of the MD simulation (the last 25 ns). The values are presented in Table 4. 

The highest free energy was identified for **SA05** (−71.23 ± 5.29 kcal/mol), close to the one of **sorafenib** (−69.39 ± 3.63 kcal/mol). **SA02** and **SA04** exhibited similar ΔG (−65.38 ± 4.13 kcal/mol and −64.21 ± 4.18 kcal/mol). Interestingly, the lowest ΔG was identified for **SA03** and with an increased standard deviation (−47.20 ± 11.74 kcal/mol) indicating an unfavorable effect due to the 4-chloro-substitution. This large change in the free energy of binding could lead to some instability of the predicted ligand–protein complex. The lowest standard deviation was identified for **sorafenib**, indicating its complex with VEGFR2 is remarkably stable over time. A low standard deviation was also identified for **SA02**, **SA04,** and **SA05,** close to that of **sorafenib**, suggesting a good binding to the VEGFR2.

The analysis of the detailed interactions between the compounds and VEGFR2 suggests that the main driving force for inhibitor binding are the van der Waals interactions, in agreement with other reports from the literature. For comparison, Wang et al. reported a similar value for this type of interaction of **sorafenib** with VEGFR2 (−60.93 ± 0.27 kcal/mol) [76]. **SA05** expressed the highest van der Waals interaction with the target (−64.37 ± 3.33 kcal/mol). For **SA03**, the high standard deviation of the energy of the van der Waals interaction stands out (−46.68 ± 10.66 kcal/mol).

The highest electrostatic interactions were identified for **sorafenib** (−39.67 ± 3.76 kcal/mol), followed by **SA05** (−33.84 ± 4.39 kcal/mol) and **SA02** (−32.95 ± 3.93 kcal/mol). **SA03** and **SA04** exhibited a significantly lower interaction with the VEGFR2 electrostatically. The same trend was observed for the energy of solvation—highest for **sorafenib** (29.47 ± 2.65 kcal/mol), followed by **SA05** (26.97 ± 3.58 kcal/mol) and **SA02** (26.06 ± 3.24). 

**Figure 8:**
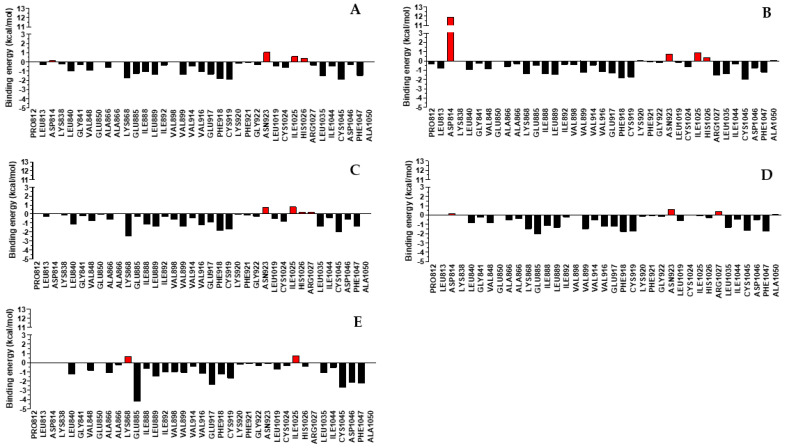
Graphical representation of free energy decomposition of the involvement of each amino acid found within 5Å during the last 25 ns of the MD simulations for: **SA02** (**A**); **SA03** (**B**); **SA04** (**C**); **SA05** (**D**); **sorafenib** (**E**).

To identify in a detailed way which are the most important amino acids for interaction with the tested compounds and **sorafenib** as a reference, an energy decomposition was performed for the evaluation of every interaction of the compounds with the amino acids found within 5Å from the ligands (Figure 8, exact values are provided in Table S1—Supplementary). Experimental data suggest a similar interaction pattern identified for the **SA02–SA05** series and **sorafenib.** A high contribution, mainly through hydrophobic interactions, was identified for a series of amino acids that reside in the region that accommodates the 4-aryloxy-quinazoline (**SA02–SA05**) and 4-aryloxy-pyridine (**sorafenib**) moieties (Glu917, Phe918, Val916, Phe1047, Val899, Leu1035). An increased contribution was also identified in the DFG-motif region (Glu885, Asp1047, Cys1045) of VEGFR2 for **sorafenib** and **SA02–SA05,** which might suggest an increased affinity for the inactive form of the receptor when Phe1047 is flipped outwards of the allosteric pocket. Important to mention is that the data suggest that the stability of the **SA02–SA05** complexes relies to a lesser extent on the already established interactions (Glu885, Asp1047) compared to **sorafenib** and they form distinctive favorable bonds with amino acids such as Lys868 (−1.38 to −2.43 kcal/mol) that are found to hinder the affinity of the **sorafenib** complex (+0.67 kcal/mol). 

Surprisingly, the energy decomposition performed for **SA03** indicates a high repulsion with Asp814 found in the allosteric pocket, composed of 5.50 kcal/mol from van der Waals and 5.37 kcal/mol from the electrostatic interaction with the sidechain of the respective amino acid and a negligible amount from the interaction with the backbone where the respective amino acid is found (data not provided). At a closer view, the negatively charged sidechain of Asp814 interacts via a salt bridge with the positively charged sidechain of Arg1027. The substituents from the proximal benzene ring are found in the proximity of sidechain Asp814 and, being electronegative and bulky, as is the chlorine atom, a repulsion can appear between the two negatively charged moieties. Thus, disruption of the salt bridge between Asp814 and Arg1027 would be an energy-demanding process from a thermodynamic point of view, with a doubtable effect on the binding of the inhibitor in the ATP-binding pocket of VEGFR2. This phenomenon is seen to a lesser extent in the case of **SA02, SA04,** and **SA05** and this could suggest that the nature and the orientation of the substituent on the distal benzene ring that occupies the hydrophobic pocket comprising the sidechains of Ile888, Leu813, and Ile1025 is critical for the VEGFR2 inhibition.

### 4.6. FMO Analysis and Chemical Reactivity Descriptors 

Quantum chemistry descriptors have been successfully used for the prediction of physicochemical properties, biological activity, and chemical reactivity in conjugated π-systems [62,77]. By extending the principle to drug discovery, the highest occupied molecular orbital (HOMO) and the lowest occupied molecular orbital (LUMO) are predictive descriptors for the electronic interactions of a compound with a biological target [78,79]. Higher HOMO and lower LUMO energy are associated with better stabilizing interactions and therefore a higher binding affinity [62]. HOMOs are electron-donor regions that can provide π-π and hydrophobic interactions within a target, while LUMOs are acting as electron-acceptor regions [80,81]. 

**Figure 9:**
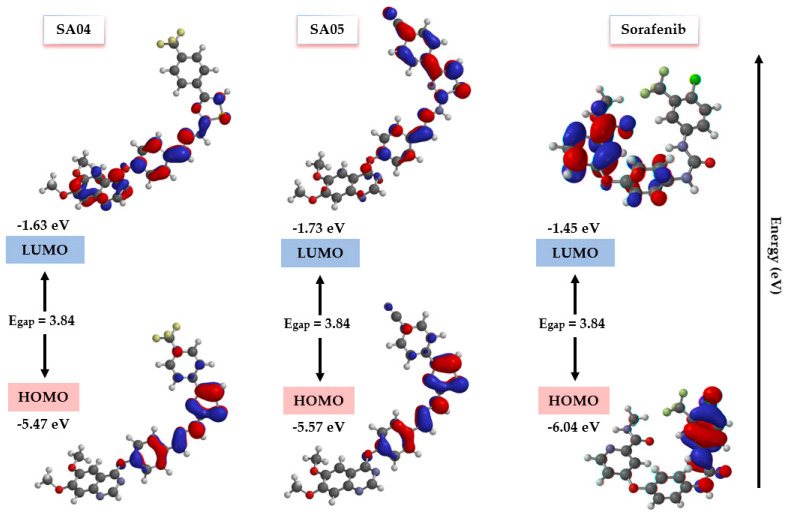
Graphical representation of molecular orbitals’ surface distribution and energy levels of HOMO and LUMO of the studied compounds **SA04, SA05,** and **sorafenib** at the B3LYB/6-311G++(d,p) level.

The 3D distribution of HOMO-LUMO orbitals of two selected representative compounds (**SA04, SA05**) and the reference drug **sorafenib** are represented in Figure 9 (for **SA01–SA03** and **SA06–SA07** see Figure S43, Supplementary). Available data suggest the importance of the complementarity between the opposite orbitals of ligand and protein to form a stable complex [82]. For compounds **SA01–SA04** and **SA06–SA07,** the HOMO orbitals follow the same pattern by occupying the electron-rich area of the molecules (thiazole ring, hydrazone moiety, and phenoxy group) while the LUMO orbitals are confined on the π-deficient quinazoline ring and extend to a different degree to the phenoxy and hydrazone region. A similar pattern is followed by **sorafenib,** with HOMO orbitals confined on the 4-chloro-3-trifluoromethyl-phenyl-urea group while the LUMO orbitals are located on the π-deficient pyridine moiety. Of particular interest is compound **SA05**, which displays a similar HOMO orbital distribution (the thiazole, hydrazone, and phenoxy groups) while the LUMO orbitals form an extended system that overlaps with the HOMO orbitals facilitating an effective charge transfer transition [83]. This phenomenon might be associated with the strong electron-withdrawing effect of the cyan group.

The frontal molecular analysis (FMO), the global reactivity descriptors (GRD) (I, A, μ, S, η, ω, N, ∆N), and the dipole momentum of the **SA01–SA07** series and **sorafenib** are depicted in Table 5. All the synthesized compounds displayed a smaller energy gap (3.42–3.84 eV) compared to **sorafenib** (4.59 eV). A large energy gap defines a “hard” molecule that is less polarized. Soft molecules have a smaller gap and are highly polarizable. Decreased chemical hardness and increased global softness are also associated with favorable charge transfer and a superior biological effect [84]. The electrophilicity index (ω = 9.68–12.79 eV) of all the novel compounds proves that they are strong electrophiles (ω >1.5 eV) capable of forming stable interactions with a biological target [85]. The weakest nucleophile is **sorafenib** (0.06 eV) followed by **SA05** (0.07 eV) which is classified as a marginal nucleophile according to Domingo’s scale [86]. Effective charge separations defined by a dipole moment were identified for **sorafenib** (6.68 D) and **SA05** (5.59 D) which could have an important role in electrostatic interactions.

Additionally, the molecular electrostatic maps of **SA01–SA07** and **sorafenib** were determined to identify the electron-rich and electron-deficient areas (Figure S44, Supplementary). These representations can provide insight into the regions susceptible to nucleophilic or electrophilic attacks and the key interactions within a biological target [87]. Regions colored in blue indicate a low electron density while red areas are rich in electrons. Increased electronic density was identified around the two basic functions of the quinazoline ring, hydrazone moiety, and electron-withdrawing groups (-F, -Cl, -CN) that substitute the lateral phenyl group.

### 4.7. Wound Healing (Scratch) Assay 

**Figure 10:**
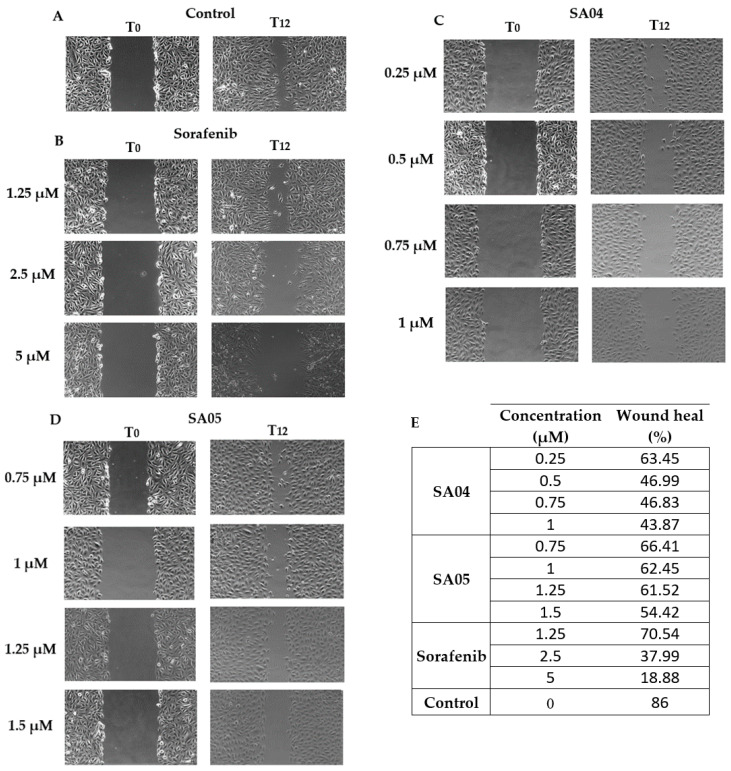
Migration of EA.hy926 cells after 12 h (T12) (**A**) in the presence of the negative control (DMSO 0.2%); (**B**) in the presence of **sorafenib**; (**C**) in the presence of **SA04**; (**D**) in the presence of **SA05**; wound heal (%) correlated with the concentration of the tested samples (**SA04, SA05, sorafenib**) (**E**).

Vascular endothelial cell migration is a key step in the development of novel blood vessels and it is highly directed by the local microenvironment [4,88]. An experimental model to assess the impact of selected compounds, **SA04, SA05**, and **sorafenib,** on EA.hy926 motility was employed to further evaluate the anti-angiogenic potential. While **SA01–SA05** provided an increased antiproliferative activity on EA.hy926 cells with minimal difference in the IC_50_ value, for the wound healing assay we chose to select **SA05**, due to the favorable in silico results that might suggest an increased affinity for the ATP-binding pocket of the inactive form of VEGFR2 and high potency towards the entire cell panel, and **SA04** due to the favorable selectivity index. The effects of **SA04, SA05**, and **sorafenib** on the chemotactic motility of EA.hy926 are provided in Figure 10. For all the evaluated compounds, the wound’s closure and the number of invasive cells decreased in a dose-dependent manner upon treatment of EA.hy926 cells with a concentration gradient for each compound (under IC_50_ value), after 12 h. The provided data confirm the negative impact of the selected compounds on vascular endothelial motility at lower doses compared to IC_50_ in a similar manner to the reference drug **sorafenib** and support the initial hypothesis of the anti-angiogenic potential of the evaluated compounds. 

### 4.8. Chorioallantoic Egg Membrane (CAM) Assay 

**Figure 11:**
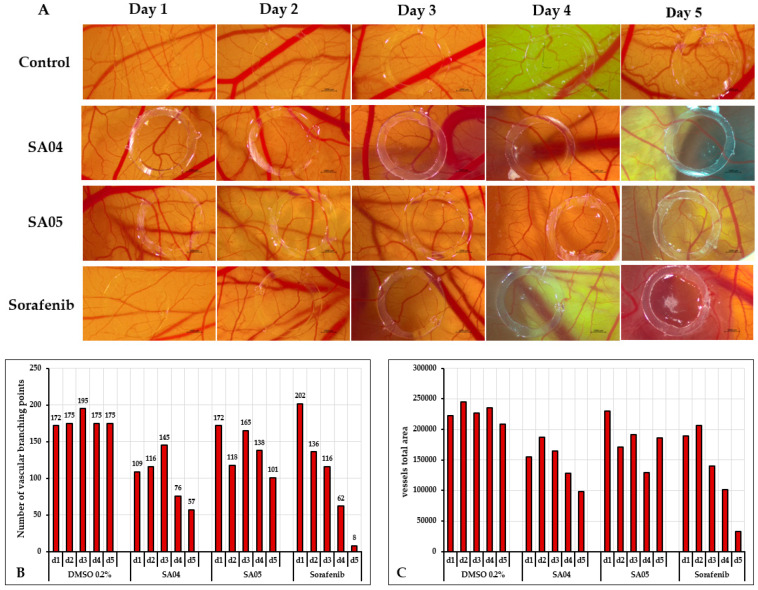
(**A**) The assessment of angiogenesis in the chick chorioallantoic membrane subjected to **SA04, SA05, and sorafenib** applications entailed the acquisition of indicative stereomicroscopic images throughout the experimental period from day 1 to day 5. The comparative control group was administered solely with the solvent (0.2% DMSO). (**B**) Quantitative assessment of the number of vascular branches and (**C**) quantitative assessment of the total vessel area with IKOSA Prism AI Cam Assay (V3.1.0) for **SA04, SA05, sorafenib,** and the control sample (DMSO 0.2%) from day 1 to day 5.

In the assessment of the anti-angiogenic potential of **SA04** and **SA05**, the compounds were administered consecutively for 5 days on the chorionic membrane, concomitantly with the solvent utilized for solution preparation (0.2% DMSO) as control sample and the positive control represented by **sorafenib** (Figure 11A).

Concerning **SA04**, angiogenesis during the initial 3-day period, marked by the germination phase, exhibited no significant alteration. The architectural arrangement of the vascular plexus within the applied region remained akin to that in the surrounding area. Between the 3rd and 5th days of the experiment, a discernible reduction in the diameter of blood vessels and vascular branching was noted. Concurrently, noteworthy alterations were observed in the vascular plexus for **SA05**, particularly on the 4th and 5th days of the sample application. In this instance, a decrement in vascular density and the quantity of capillaries within the vascular ring was evident. 

As anticipated, notable alterations in the angiogenic process were evident in the context of the **sorafenib**, manifesting considerable reductions in both the dimensions and quantity of vascular capillaries. By the 5th day of the experiment, a reticular pattern was discerned within the vascular ring, unequivocally signaling the inhibition of the angiogenic process. It is important to highlight that throughout the experimental duration, no manifestations of vascular irritation, such as hemorrhage, lysis, or vascular coagulation, were discerned in any of the examined samples (Figure 11A). 

A quantitative assessment of the anti-angiogenic potential of **SA04, SA05,** and **sorafenib** compared to the control sample (0.2% DMSO) was achieved using the IKOSA Prisma AI CAM assay (Figure 11B,C). In the germination phase, for both **SA04** and **SA05**, a gradual increase in the number of vascular branching points was identified from day 1 to day 3. The same pattern was observed in the control sample, with the mention that the peak number of vascular branching points from day 3 is 1.3-fold lower for **SA04** and 1.18-fold lower for **SA05**. A marked decrease in the vascular branching was identified on day 4 (47% reduction) and day 5 (60% reduction) compared to day 3 for **SA04** and to a lesser extent for **SA05** on day 4 (16% reduction) and day 5 (39% reduction) compared to day 3. Overall, on day 5, for both **SA04** (67% reduction) and **SA05** (42% reduction), a significant reduction in the number of the vascular branching points was identified compared to day 5 of the control sample (0.2% DMSO). For **sorafenib**, a gradual decrease was identified during the experiment (day 2–day 5). The same trend was also identified for the vessels’ total area. It is important to outline that during the experiment only **SA04** displayed a consistent reduction in the number of vessels compared to **SA05**, and to a lesser extend compared to the reference drug **sorafenib**.

### 4.9. In Silico Physicochemical and Pharmacokinetics Prediction 

The physicochemical and pharmacokinetic parameters of the synthesized compounds **SA01–SA07** and **sorafenib** studied with the Swiss ADME web tool are depicted in Table S2 (Supplementary). Lipinski’s rule of 5 (RO5) describes a series of parameters pivotal for the pharmacokinetics of potential novel drugs with favorable oral absorption [89]. Compounds **SA01–SA05** displayed only one violation of the RO5, having a higher than 500 g/mol molecular weight (Mw), while **SA06** and **sorafenib** displayed no violations. Unfavorable results were recorded for **SA07**, which displayed two violations of LR5 and one violation of Veber’s rule, due to increased topological polar surface area (>140 Å²) [90]. Overall, most compounds displayed superior lipophilicity and slightly lower solubility compared to **sorafenib**, all the evaluated compounds (including **sorafenib**) being classified as “poorly soluble” or “moderately soluble” with low gastrointestinal absorption. A better insight into the oral absorption of the compounds can be provided by the graphical representation of the bioavailability radar (Figure S44, Supplementary). Different approaches have been employed to address the low solubility of **sorafenib** (formation of tosylate salts, nano-delivery systems) that could also address the issue for the novel compounds [91,92]. 

## 5. Conclusions 

In summary, seven novel quinazoline–thiazole hybrids (**SA01–SA07**) were obtained through scaffold hopping, successfully synthesized, and evaluated for their antiproliferative and anti-angiogenic potential. Remarkable results were obtained for selected compounds (**SA02–SA05**) that displayed significantly higher antiproliferative activity (IC_50_ =1.83–4.24 µM) compared to reference drug **sorafenib** on HepG2 cells. The 4-phenyl substitution was identified as pivotal for the potency of the compounds with the 4-methyl-thiazole derivative **SA06** displaying no activity at all and low solubility. EWGs 4-substituted derivatives (**SA02–SA05**) displayed significantly higher antiproliferative activity compared to EDGs 4-substituted compounds (**SA01, SA07**) and up to 3.5-fold higher compared to the reference drug **sorafenib** for the most active compound **SA05** on HepG2 cells. 

Considering the scaffold hopping approach for the development of the series **SA01-SA07**, we further evaluated the affinity for the kinase domain of the human VEGFR2 through various in silico methods. An increased similarity between the posing of the compounds and the reference drug **sorafenib** within the active site of VEGFR2 was identified through molecular docking. Available data suggest that the four main pharmacophores (main core, lipophilic linker, hydrophilic group of the DFG-region, marginal hydrophilic group) of both **sorafenib** and **SA01–SA07** can successfully accommodate the four distinct regions of the catalytic unit. Moreover, the 4-substituted thiazole moiety dives deeper into the allosteric pocket of VEGFR2, which might provide a distinctive binding mode compared to the reference drug **sorafenib**. It is important to outline that all the evaluated compounds and **sorafenib** displayed at least three H-bonds (Cys919, Glu885, Asp1046) within the hinge region and the DFG region. An overview of the MDs can outline that the same compounds that displayed an increased antiproliferative potential on HepG2 cells (4-phenyl-EWG substituted compounds, **SA02–SA05**) also formed the most stable complexes with the kinase domain of VEGFR2, with the most favorable results being reported for the 4-cyano-phenyl derivative **SA05** for both evaluations. MM-PBSA free energy calculation provided a better view of the nature of the interactions within the most stable complexes (**SA02–SA05**, **sorafenib,** and VEGFR2). Energy decomposition of the amino acids found within 5Å from the ligands led to the conclusion that a similar binding pattern is employed for both **sorafenib** and series **SA02–SA05**. For the synthesized series, however, of particular concern is the disruption of the Asp816–Arg1047 salt bridge that negatively impacts the stability of the complexes. Additionally, the evaluation of the quantum chemistry descriptors led to interesting results that could partially explain the high stability of the **SA05**–VEGFR2 complex. 

The anti-angiogenic potential of the compounds was assessed by quantifying their impact on the proliferation, migration, and differentiation of vascular cells. Six out of the seven compounds displayed a significant antiproliferative activity on EA.hy296 cells compared to reference drug **sorafenib** in the range of 0.79–5.85 µM. The same SAR principles were outlined similarly to HepG2 and the in silico evaluation. Skin toxicity evaluation, by the mean of IC_50_ assessment on BJ cells, led to the identification of **SA04** as the most selective compound.

The available data led to the selection of **SA04** and **SA05** for the further evaluation of the potential to inhibit vascular cell migration (wound healing assay) and differentiation (CAM assay). Both compounds displayed an increased negative impact on the vascular cells’ motility in a concentration-dependent manner after 12 h with comparable results to that of the reference drug **sorafenib**. The studied compounds displayed visible inhibition of both the total number of vascular branching points and total vascular area after 5 days of treatment in the CAM model, confirming the initial hypothesis of an anti-angiogenic effect. A possible explanation for the lower *in* ovo potential of the compounds compared to s**orafenib** could be related to the unfavorable pharmacokinetics and low solubility.

We strongly believe that the provided structural design and the original chemical framework of the series **SA01–SA07** can provide a valuable starting point for the development of powerful anti-angiogenic compounds through the means of the lead optimization process. The primary SAR observations outlined in this work, along with the already established state of the art in the field of VEGFR2 inhibitors and anti-angiogenic derivatives can provide powerful tools for the development of pre-clinical and clinical candidates.

## Figures and Tables

**Table 1:** Calculated IC_50_ values (µM) after the exposure of EA.hy926, HepG2, and BJ cells to the synthesized compounds and **sorafenib** for 48 h and the selectivity indexes.

Comp.	Evaluated Cell Lines			Selectivity Index	
	EA.hy926	HepG2	BJ	^1^ SI_a_	^2^ SI_b_
**SA01**	**3.64**	31.20	36.25	**9.96**	1.16
**SA02**	**0.93**	**2.34**	2.35	2.53	1.00
**SA03**	**0.79**	**4.24**	2.84	3.59	0.67
**SA04**	**0.98**	**2.72**	6.79	**6.93**	**2.50**
**SA05**	**1.40**	**1.83**	2.67	1.91	1.46
**SA06**	>10	>100	>100	-	-
**SA07**	**5.85**	27.30	23.76	4.06	0.87
**Sorafenib**	**6.62**	**6.28**	**13.24**	**2.00**	**2.11**

^1^ SI_a_ = IC_50_ BJ/IC_50_ EA.hy296; ^2^ SI_b_ = IC_50_ BJ/IC_50_ HepG2.

**Table 2:** MD parameters evaluated for **SA01–SA07** and **sorafenib** in complex with VEGFR2.

System Evaluated	RMSD—Protein (nm) ^1^	RMSF Amino Acid αCarbons (nm) ^2^	RMSF Amino Acid Sidechains (nm) ^3^	RMSD—Ligand (nm) ^4^	Rg Protein(nm) ^5^	NoHB Protein–Ligand (no/ns) ^6^
apo VEGFR2 receptor + SA01	0.22	0.11	0.17	0.17	2.07	0.98
apo VEGFR2 receptor + SA02	0.21	0.12	0.17	0.10	2.06	0.91
apo VEGFR2 receptor + SA03	0.16	0.10	0.15	0.13	2.04	0.94
apo VEGFR2 receptor + SA04	0.23	0.11	0.16	0.10	2.06	0.98
apo VEGFR2 receptor + SA05	0.19	0.10	0.16	0.10	2.05	0.99
apo VEGFR2 receptor + SA06	0.31	0.09	0.14	0.19	2.07	0.98
apo VEGFR2 receptor + SA07	0.23	0.16	0.21	0.15	2.03	1.60
apo VEGFR2 receptor + SFN	0.18	0.13	0.17	0.12	2.05	1.00

^1^ The root mean square deviation of the backbone of the VEGFR2 receptor (RMSD—protein), ^2,3^ root mean square fluctuation (RMSF) of the sidechains of the amino acids from the protein and the alpha carbon from amino acids, respectively, ^4^ the root mean square deviation of the heavy atoms of the ligands (RMSD—ligand), ^5^ the radius of gyration of the VEGFR2 receptor (Rg), ^6^ the average number of H-bonds (NoHB) between the ligand and the VEGFR2 receptor in the systems evaluated in the molecular dynamics study.

**Table 3:** Decomposition of the H-bonds generated in the complexes formed by **SA01–SA07** and **sorafenib** with VEGFR2 during the 100 ns simulations and the encounter frequency (%).

	Number of H-Bond Interactions (% of Total Interactions)								
	Lys868	Glu885	Cys919	Cys1024	Ile1025	His1026	Arg1027	Asp1046	Total H-Bonds
**SA01**	18 (0.18%)	6959 (69.59%)	1762 (17.62%)	-	194 (1.94%)	-	-	882 (8.82%)	9815
**SA02**	18 (0.20%)	6605 (66.05%)	1525 (15.25%)	-	-	-	2 (0.02%)	946 (9.46%)	9096
**SA03**	20 (0.21%)	7066 (70.66%)	1300 (13.00%)	-	1 (0.01%)	-	137 (1.37%)	839 (8.39%)	9363
**SA04**	3 (0.03%)	7556 (75.56%)	1275 (12.75%)	3 (0.03%)	20 (0.20%)	-	10 (0.10%)	876 (8.76%)	9743
**SA05**	5 (0.05%)	7329 (73.29%)	1389 (13.75%)	-	88 (0.88%)	-	11 (0.11%)	1082 (10.82%)	9904
**SA06**	56 (0.57%)	7493 (74.93%)	1590 (15.75%)	-	-	-	-	669 (6.69%)	9808
**SA07**	4 (0.02%)	7355 (73.55%)	1886 (18.86%)	-	5288 (52.88%)	-	495 (4.95%)	1011 (10.11%)	16039
**Sorafenib**	-	3825 (38.25%)	178 (1.78%)	13 (0.13%)	-	13 (0.13%)	-	5833 (58.33%)	9866

**Table 4:** The free energy of binding of compounds to VEGFR2 and its decomposition in types (kcal/mol).

Compound	ΔG (±SD *)	van der Waals (±SD *)	Electrostatic (±SD *)	Solvation (±SD *)
**SA02**	−65.38 ± 4.13	−58.48 ± 3.26	−32.95 ± 3.93	26.06 ± 3.24
**SA03**	−47.20 ± 11.74	−46.68 ± 10.66	−24.37 ± 3.94	23.84 ± 3.65
**SA04**	−64.21 ± 4.18	−58.87 ± 3.29	−27.99 ± 4.33	22.66 ± 3.45
**SA05**	−71.23 ± 5.29	−64.37 ± 3.33	−33.84 ± 4.39	26.97 ± 3.58
**sorafenib**	−69.39 ± 3.63	−59.19 ± 2.81	−39.67 ± 3.76	29.47 ± 2.65

* Standard deviation.

**Table 5:** DFT calculations for the series of quinazoline–thiazole hybrids, **SA01-SA07,** and **sorafenib**.

FMO Analysis	SA01	SA02	SA03	SA04	SA05	SA06	SA07	Sorafenib
HOMO (eV)	−5.08	−5.3	−5.37	−5.47	−5.57	−5.26	−5.22	−6.04
LUMO (eV)	−1.5	−1.55	−1.95	−1.63	−1.73	−1.49	−1.54	−1.45
Energy gap (eV)	3.58	3.75	3.42	3.84	3.84	3.77	3.68	4.59
**Chemical reactivity descriptors**								
Ionization potential (I) (eV)	5.08	5.3	5.37	5.47	5.57	5.26	5.22	6.04
Electronic affinity (A) (eV)	1.5	1.55	1.95	1.63	1.73	1.49	1.54	1.45
Chemical potential (μ) (eV)	−3.29	−3.42	−3.66	−3.55	−3.65	−3.37	−3.38	−3.74
Chemical hardness (η) (eV)	1.79	1.87	1.71	1.92	1.92	1.88	1.84	2.29
Global softness (S) (eV)	0.89	0.93	0.85	0.96	0.96	0.94	0.92	1.14
Electrophilicity index (ω)(eV)	9.68	10.99	11.45	12.09	12.79	10.73	10.51	16.09
Nucleophilicity index (N) (eV^−1^)	0.10	0.09	0.08	0.08	0.07	0.09	0.09	0.06
Additional electronic charges (∆N)	1.83	1.82	2.14	1.84	1.90	1.79	1.83	1.63
Dipole moment (Debye)	2.96	2.71	3.06	3.74	5.59	2.84	5.35	6.68

## Data Availability

No new data were created or analyzed in this study. Data sharing is not applicable to this article.

## Supplementary Materials

The following supporting information can be downloaded at: https://www.mdpi.com/article/10.3390/biom14020218/s1, Figures S1–S9. The IR spectra of intermediates (1,2) and **SA01–SA07**; Figures S10–S18. The MS spectra of intermediates (1,2) and **SA01-SA07**; Figures S19-S27. The *^1^H-NMR* spectra of intermediates (1,2) and SA01-SA07; Figures S28–S36. The *^13^C-NMR* spectra of intermediates (1,2) and **SA01–SA07**; Figures S37 and S38. Cytotoxic effect of SA01-SA03 and **SA06–SA07** after a 48 h exposure of EA.hy926, HepG2, and BJ cells; Figures S39–S42. Graphical representation of the molecular dynamics parameters during the 100 ns simulation for **SA01–SA03** and **SA06–SA07**; Figure S43. Graphical representation of molecular orbitals’ surface distribution and energy levels of HOMO and LUMO of the studied compounds **SA01–SA03 and SA06–SA07**; Figure S44. Graphical representation of the molecular electrostatic map (MEP) of **SA01–SA07** and the reference drug sorafenib; Figure S45. The bioavailability radar for the **SA01–SA07** series and sorafenib generated by SwissADME; Table S1. Energetic decomposition for the amino acids found within 5Å from the ligands (kcal/mol); Table S2. In silico ADME profile and drug-likeness of the **SA01–SA07** series and **sorafenib** generated by SwissADME.
