# Supplementary material for: Discovery of A Novel Series of Quinazoline–Thiazole Hybrids as Potential Antiproliferative and Anti-Angiogenic Agents
Source: Biomolecules. 2024 Feb 12;14(2):218. doi: 10.3390/biom14020218 (PMC10886515; doi:10.3390/biom14020218)
Supplement: Supplementary file 1 [file biomolecules-14-00218-s001.zip › biomolecules-2877210-supplementary.pdf]

Article

Supplementary Material

1. Figures

1.1 IR spectrum

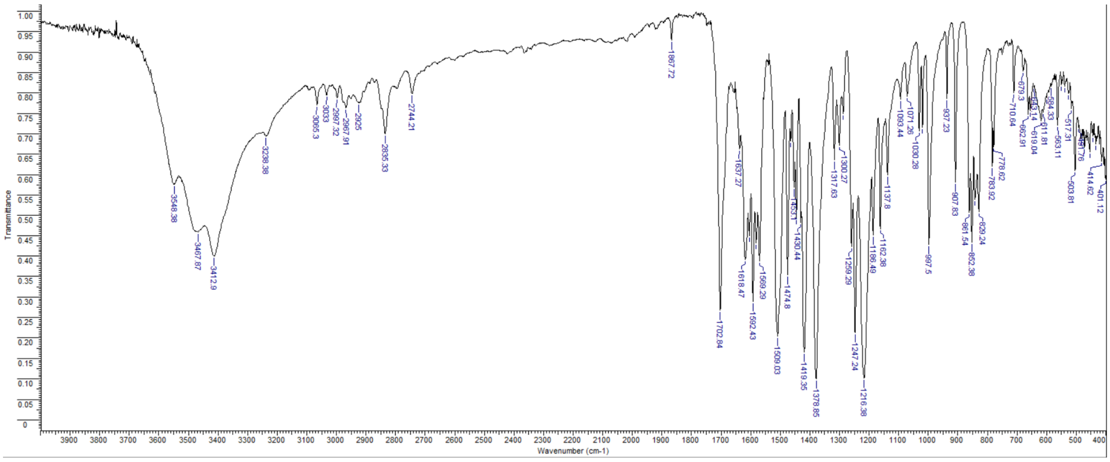

Figure S1. The IR spectrum of intermediate (**1**)

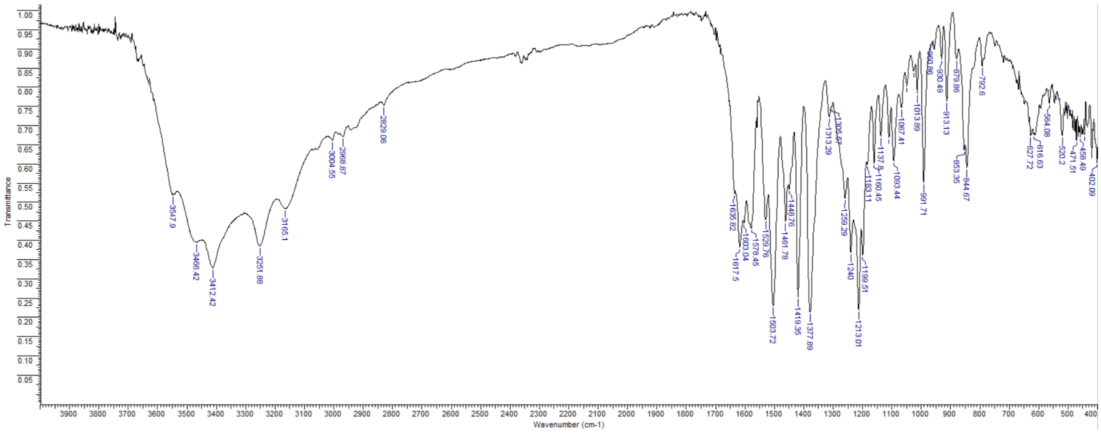

Figure S2. The IR spectrum of intermediate (**2**)

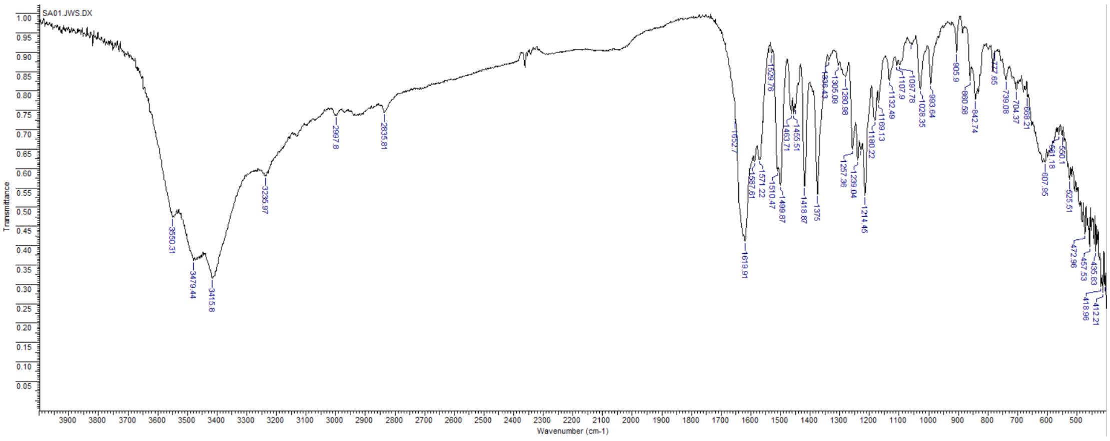

Figure S3. The IR spectrum of Compound SA01

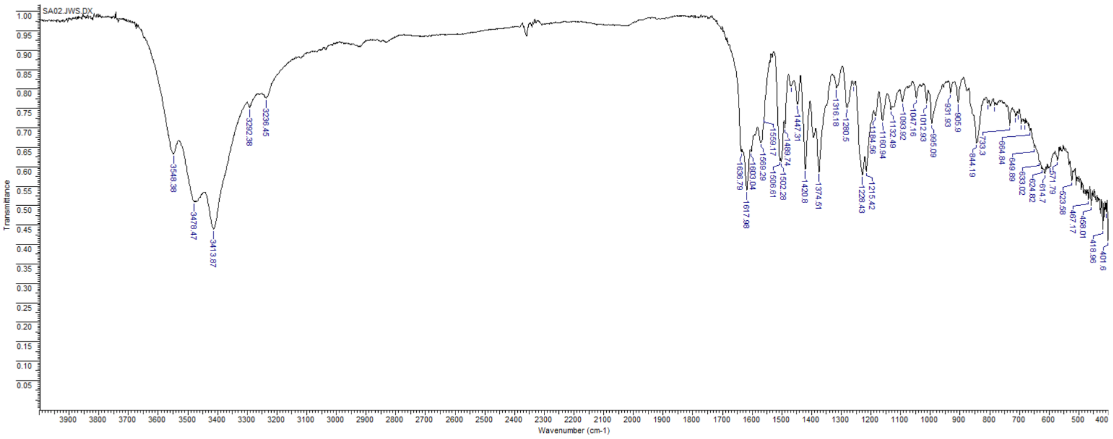

Figure S4. The IR spectrum of Compound SA02

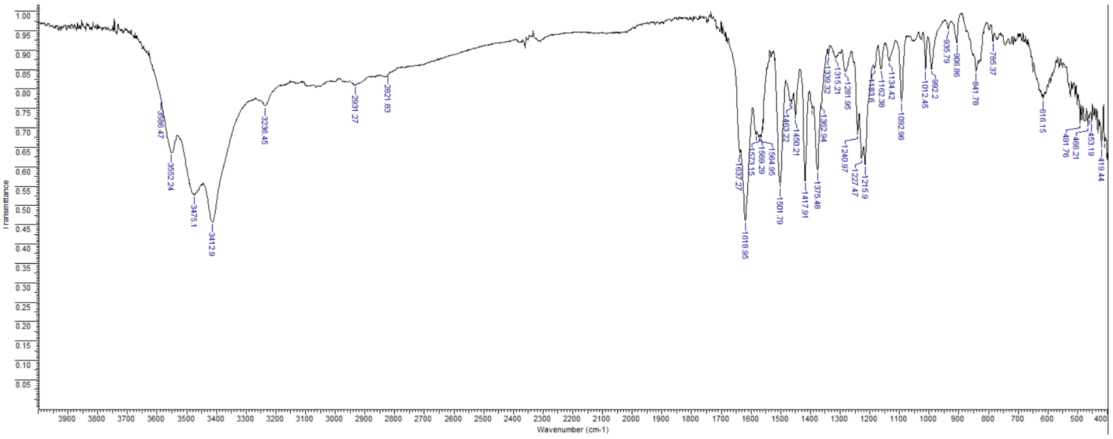

Figure S5. The IR spectrum of Compound SA03

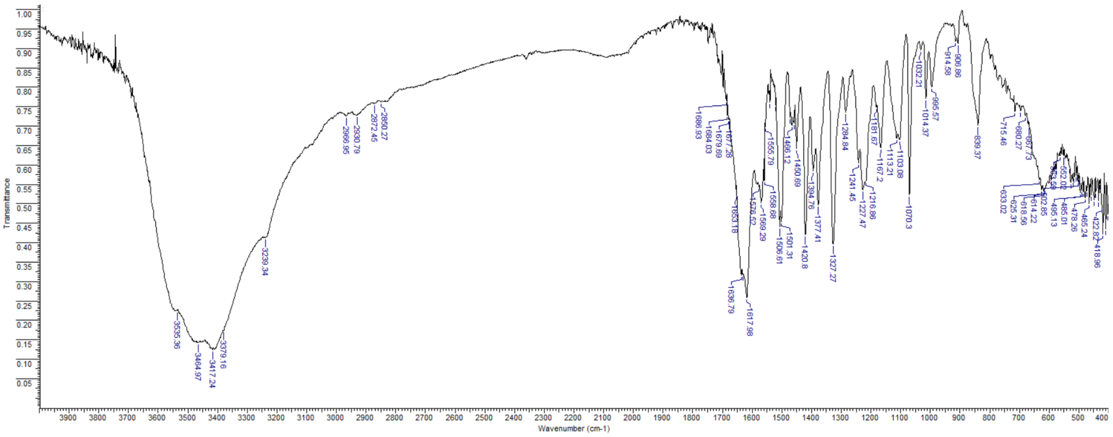

Figure S6. The IR spectrum of Compound SA04

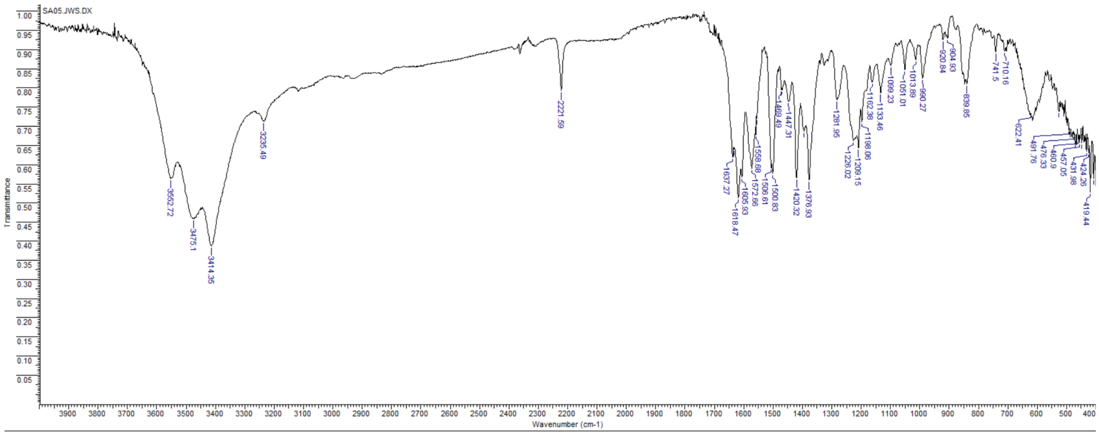

Figure S7. The IR spectrum of Compound SA05

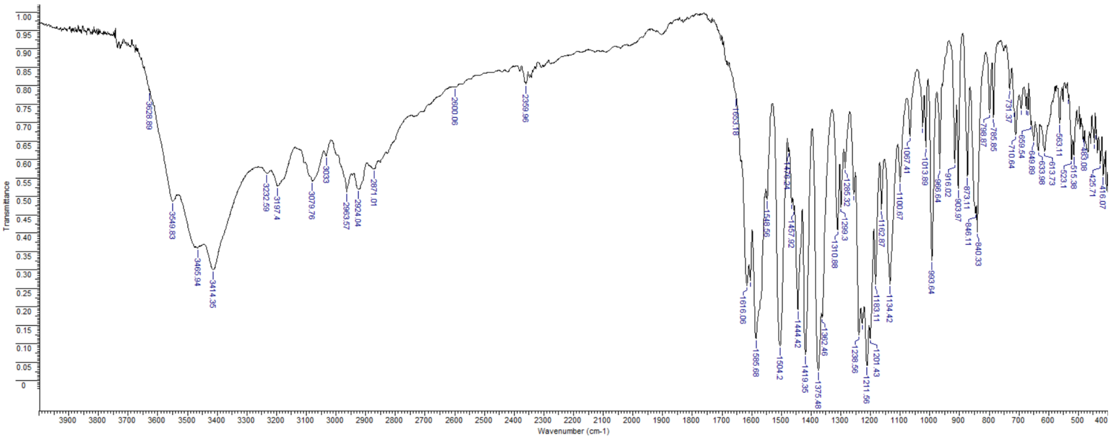

Figure S8. The IR spectrum of Compound SA06

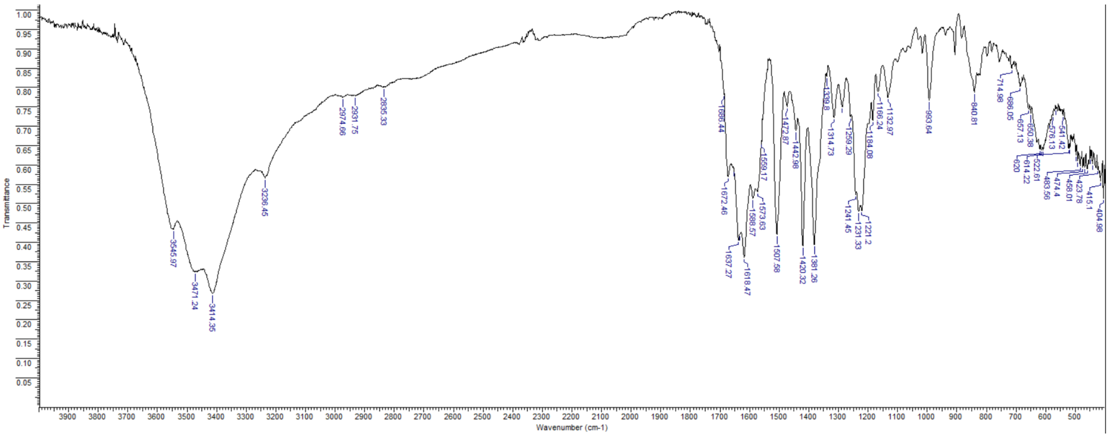

Figure S9. The IR spectrum of Compound SA07

1.2. The MS spectra

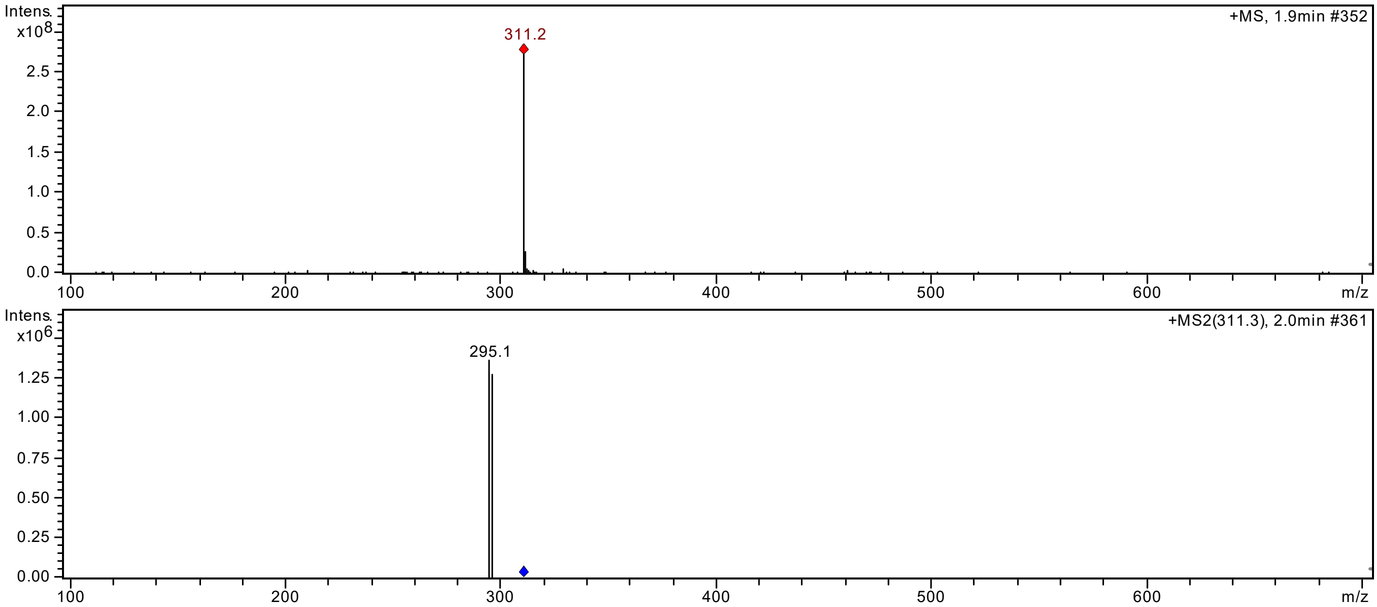

Figure S10. The MS spectra of intermediate (1)

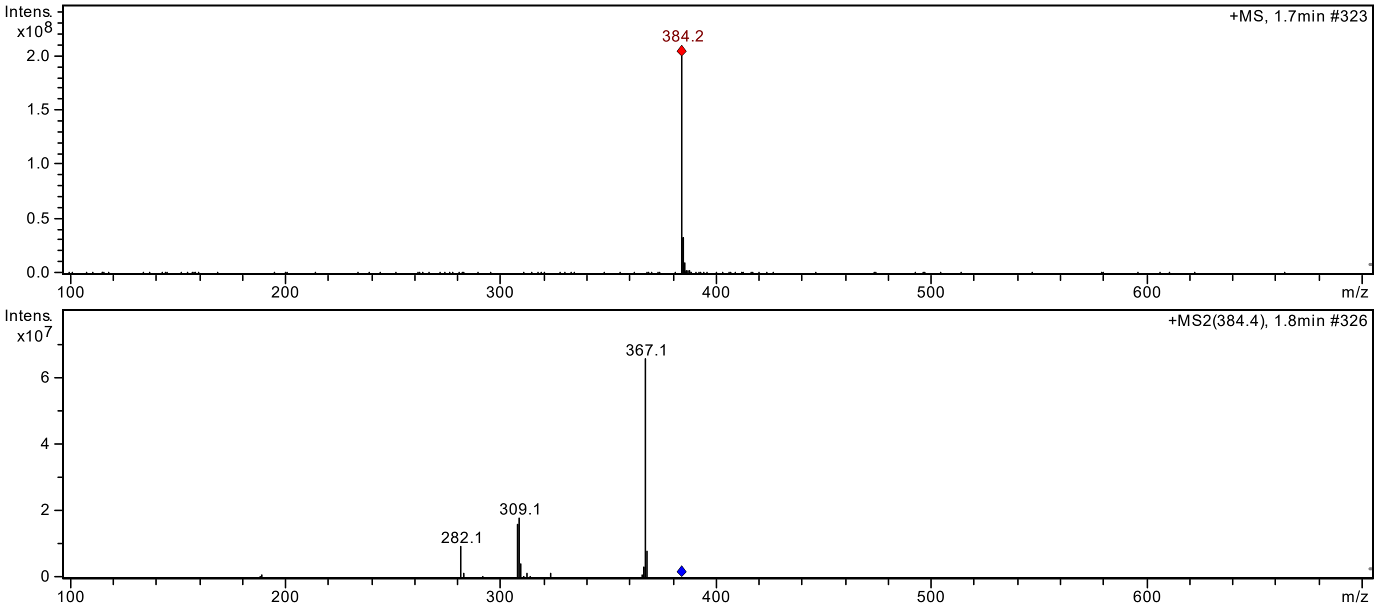

Figure S11. The MS spectra of intermediate (2)

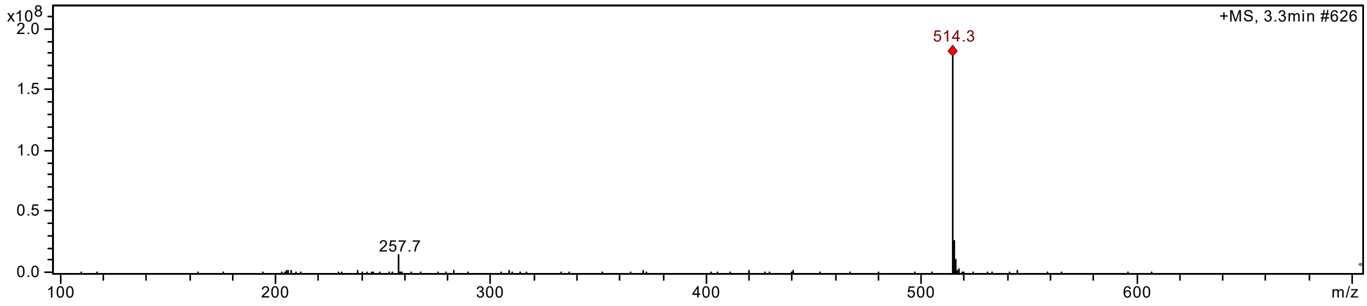

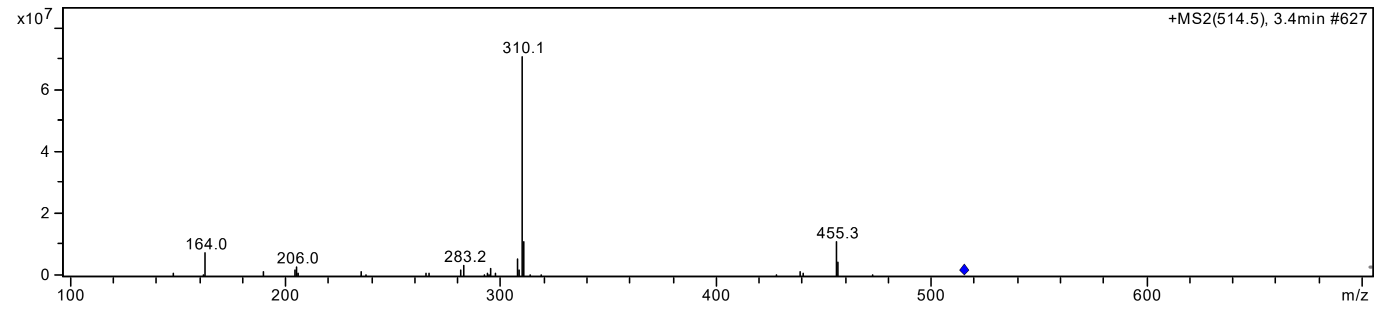

Figure S12. The MS spectra of Compound SA01

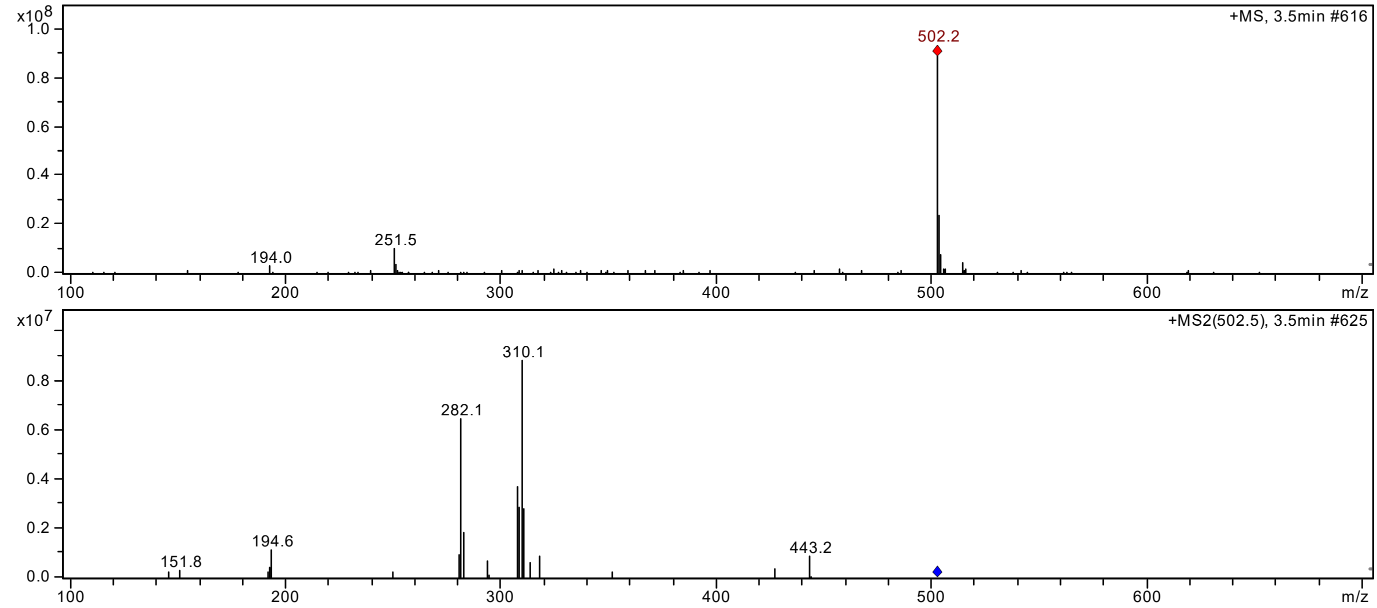

Figure S13. The MS spectra of Compound SA02

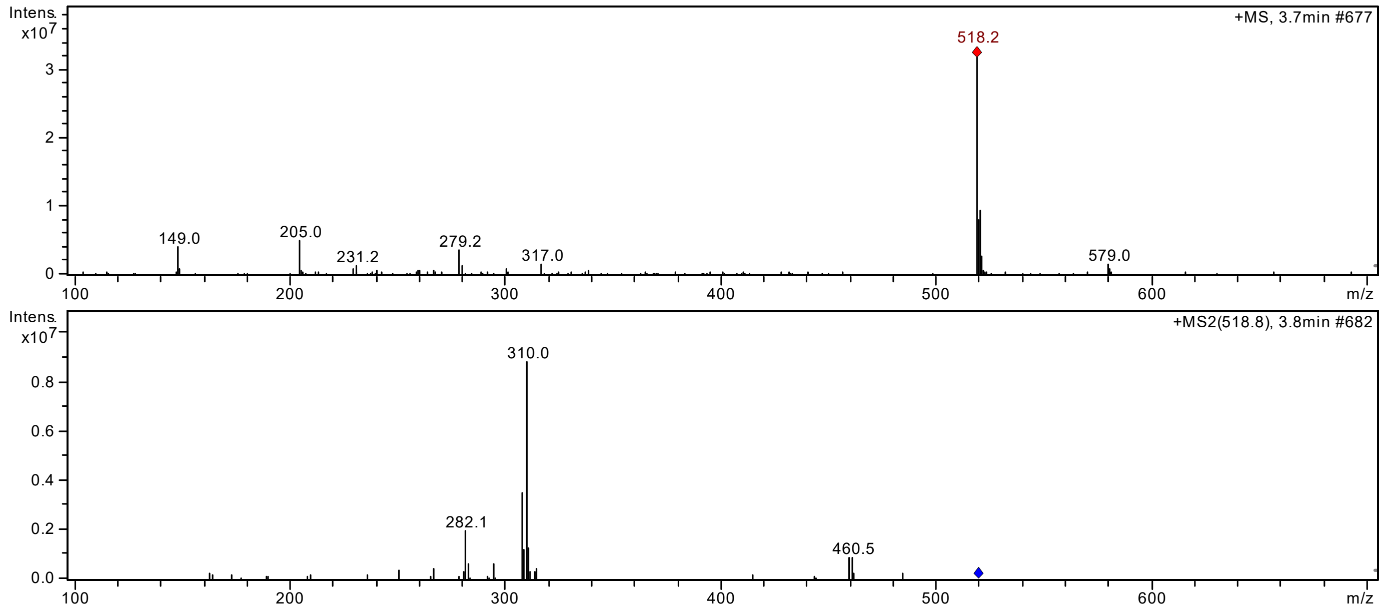

Figure S14. The MS spectra of Compound SA03

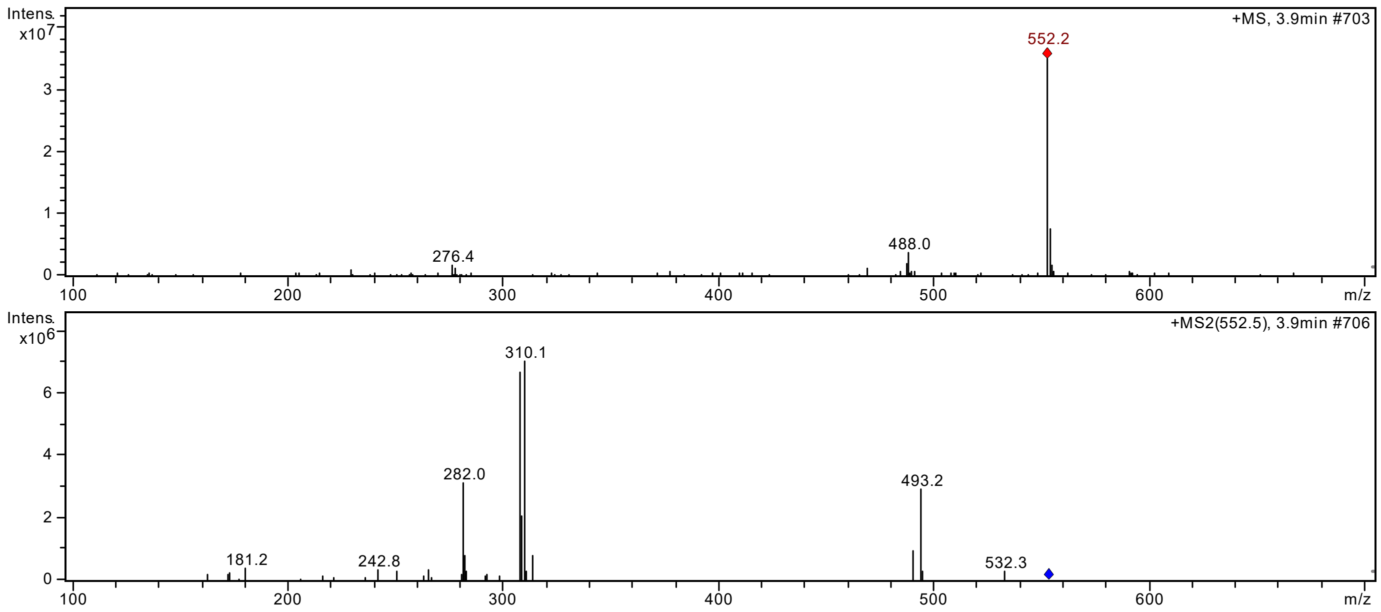

Figure S15. The MS spectra of Compound SA04

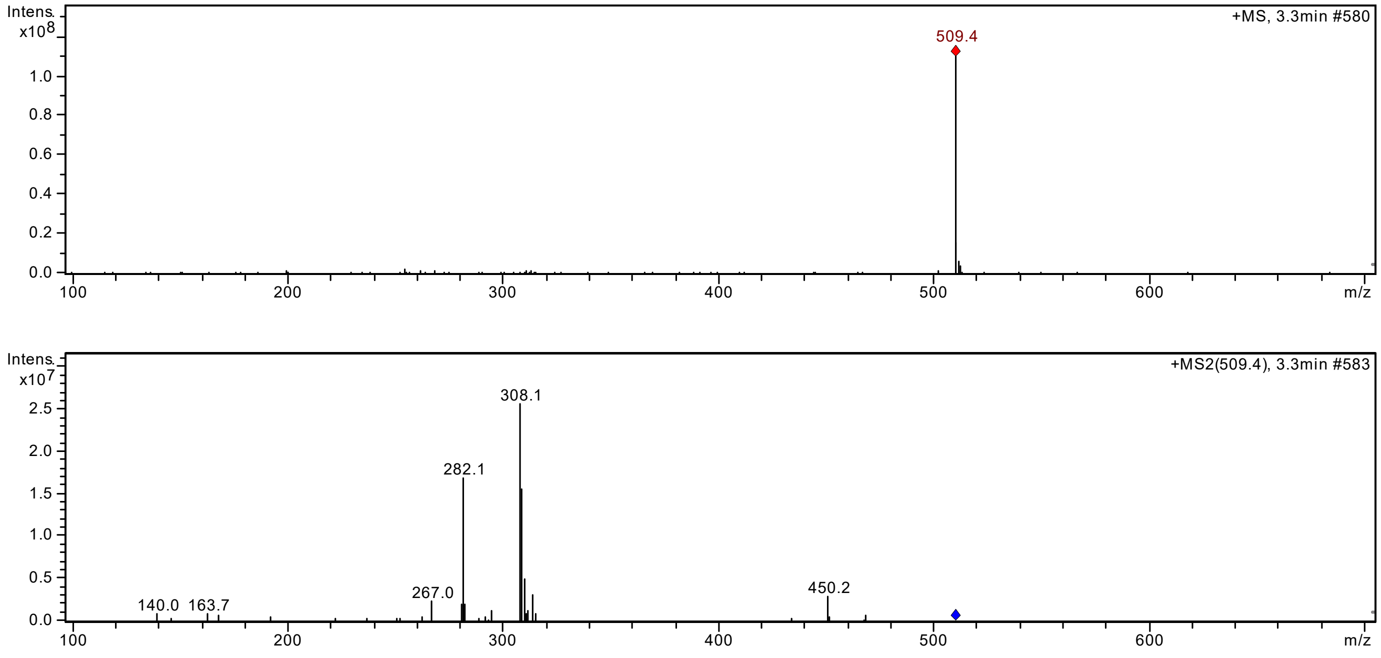

Figure S16. The MS spectra of Compound SA05

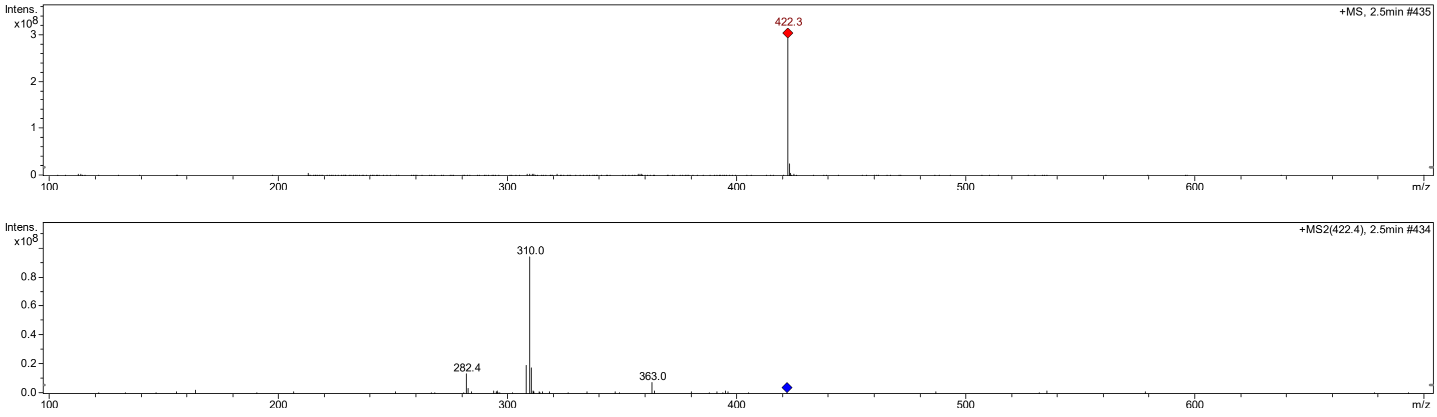

Figure S17. The MS spectra of Compound SA06

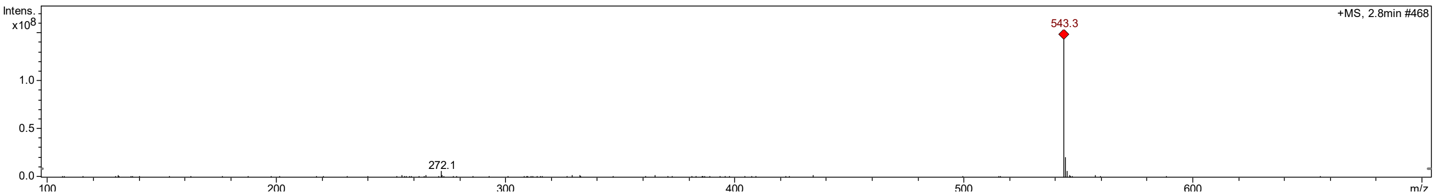

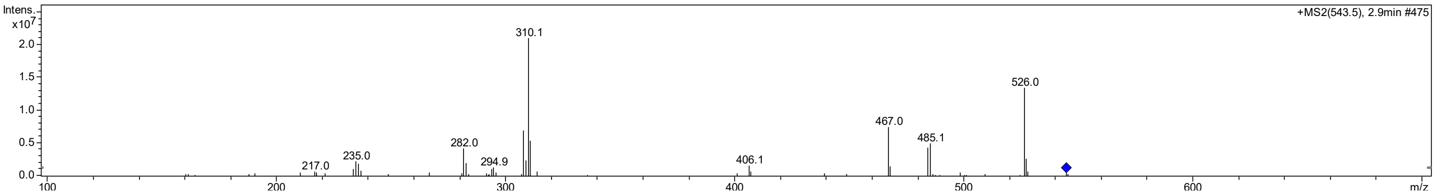

Figure S18. The MS spectra of Compound SA07

1.3. The ^{1}H-NMR spectrum

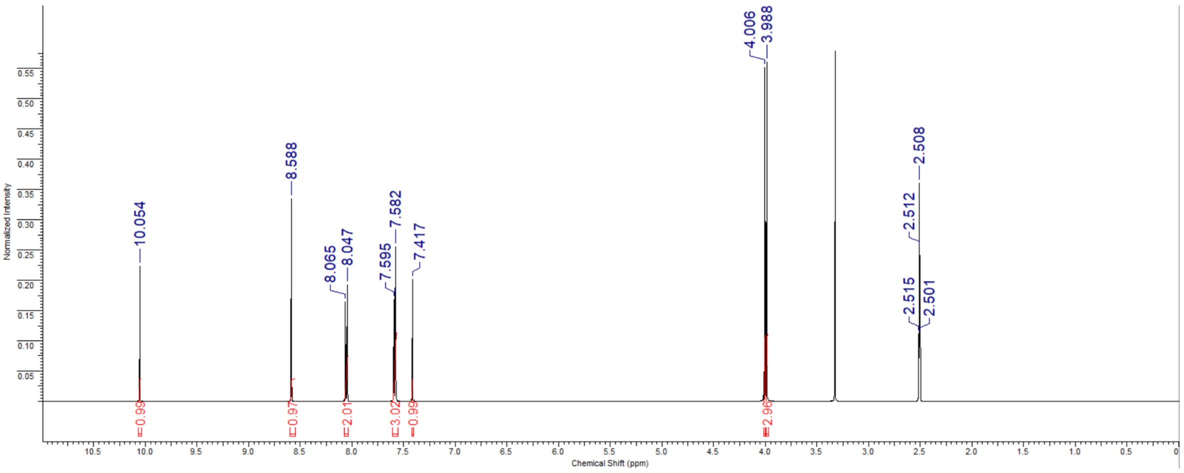

Figure S19. The *1H-NMR* spectrum of intermediate (1)

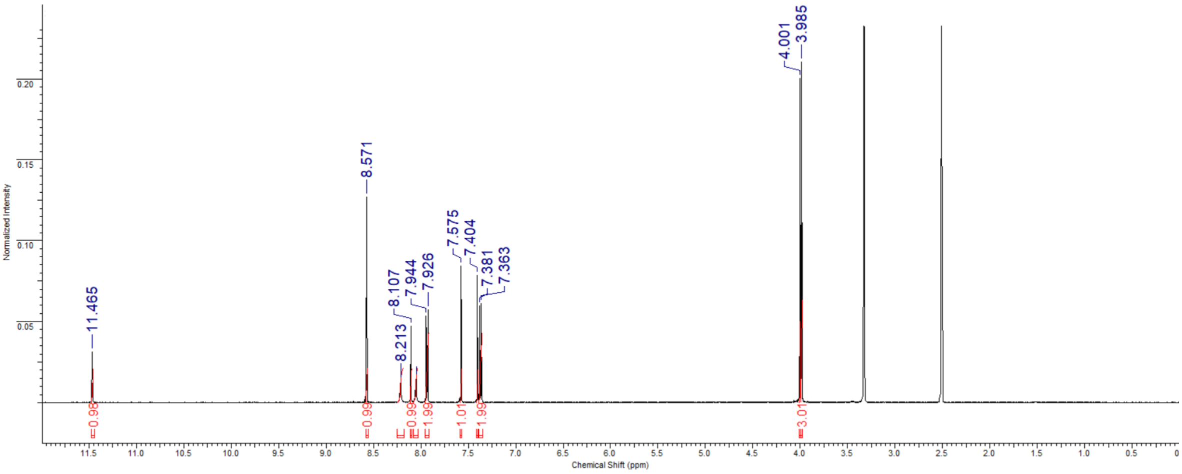

Figure S20. The *1H-NMR* spectrum of intermediate (2)

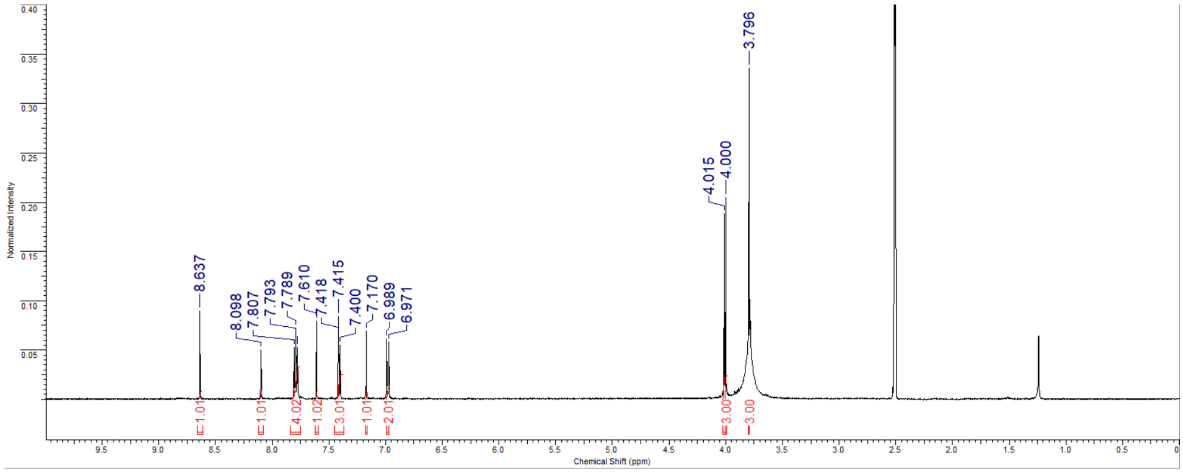

Figure S21. The *1H-NMR* spectrum of Compound SA01

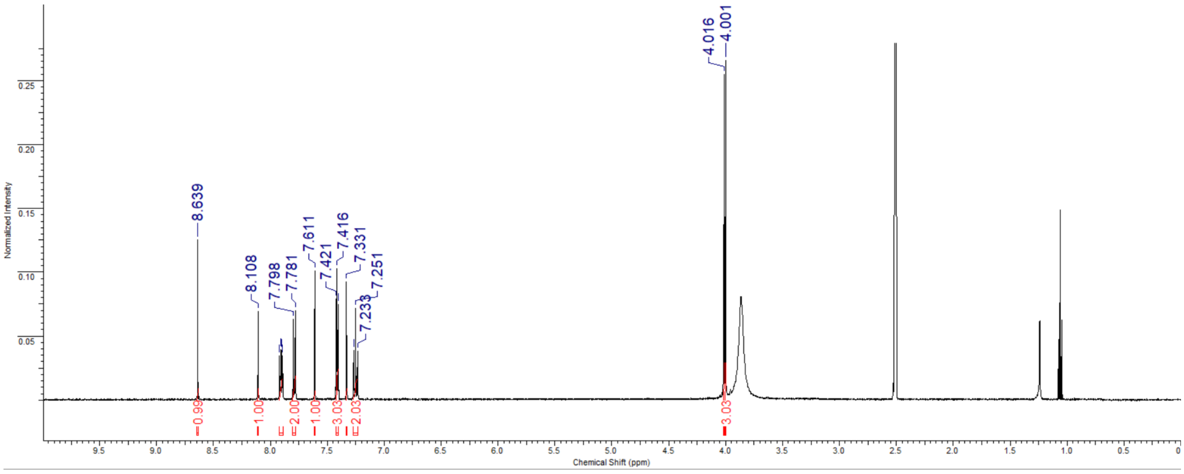

Figure S22. The *1H-NMR* spectrum of Compound SA02

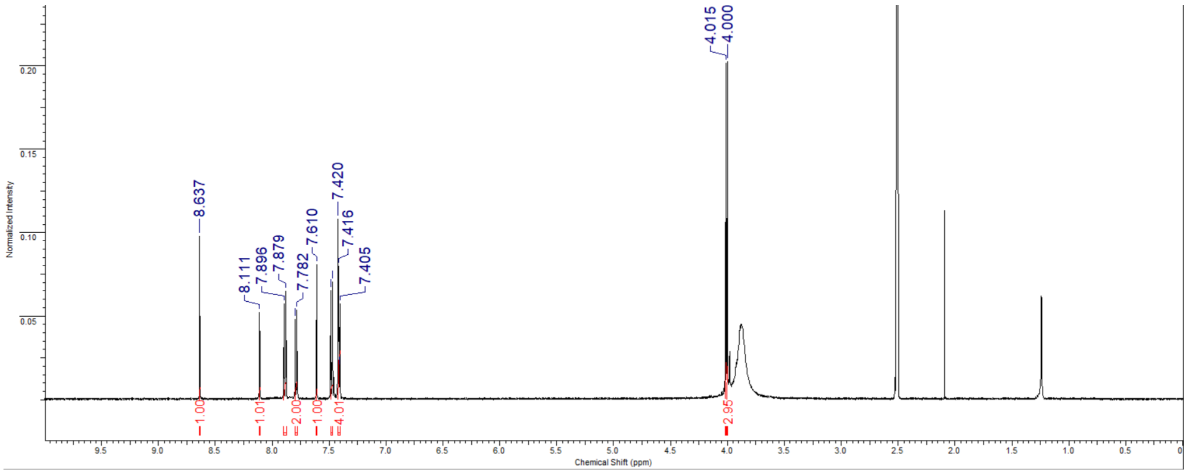

Figure S23. The *1H-NMR* spectrum of Compound SA03

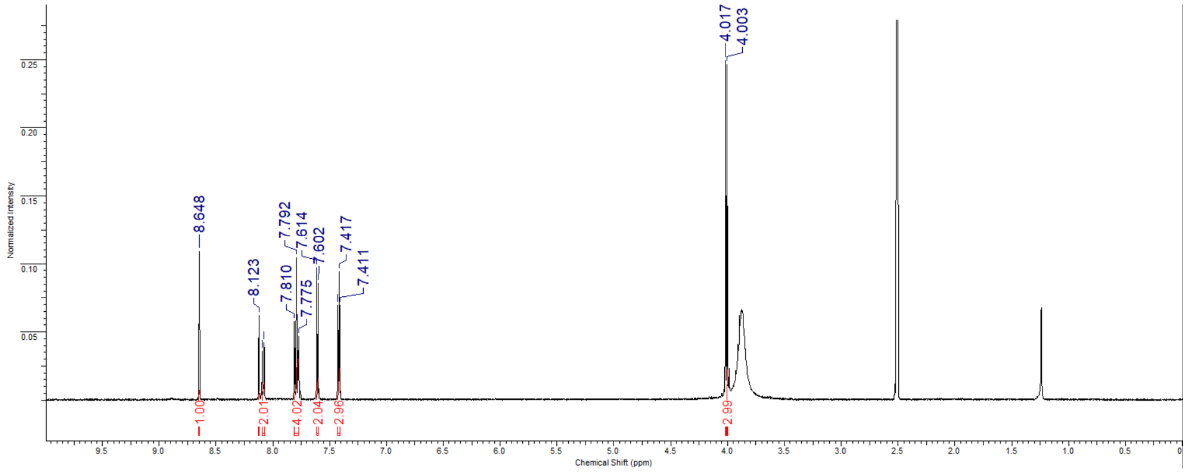

Figure S24. The *1H-NMR* spectrum of Compound SA04

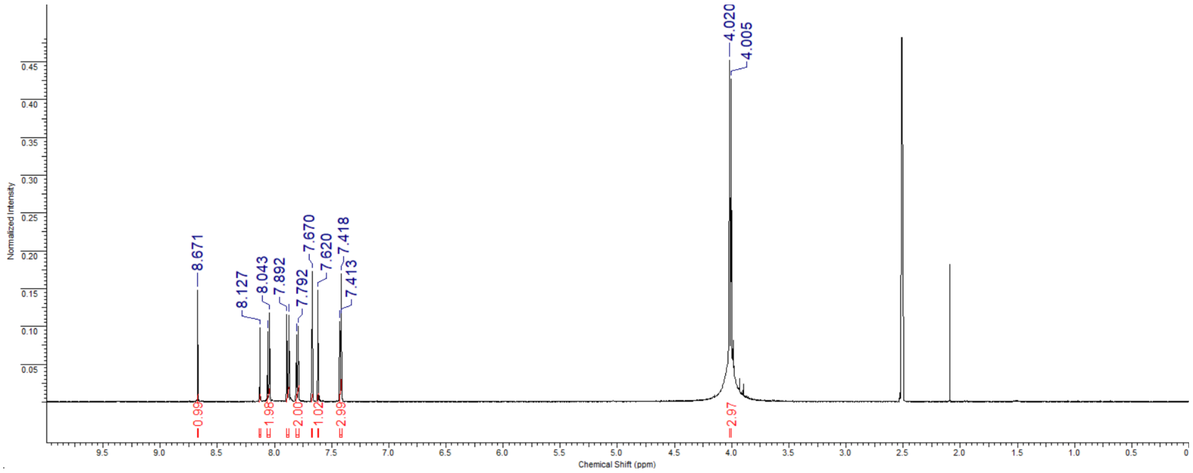

Figure S25. The *1H-NMR* spectrum of Compound SA05

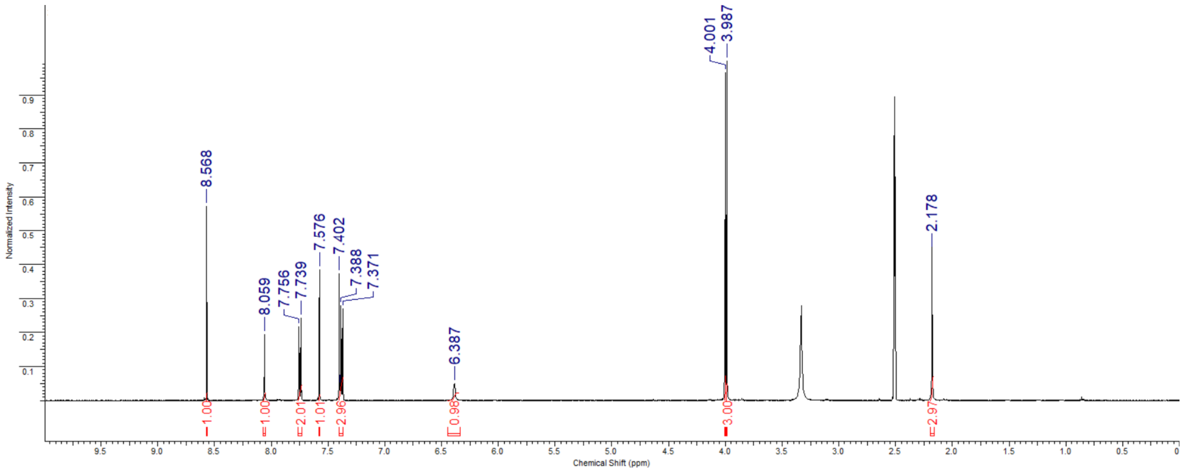

Figure S26. The *1H-NMR* spectrum of Compound SA06

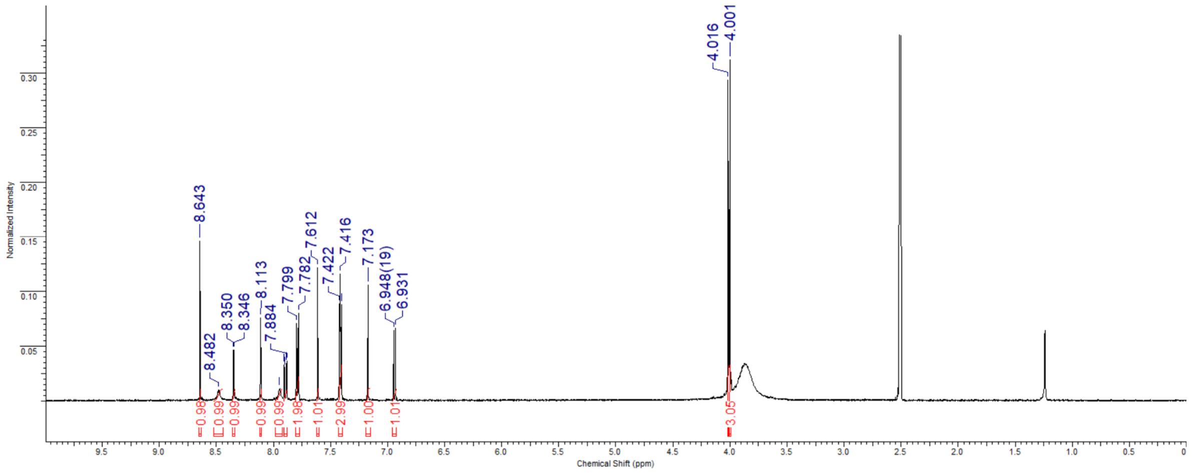

Figure S27. The *1H-NMR* spectrum of Compound SA07

1.4. The ^{13}C-NMR spectra

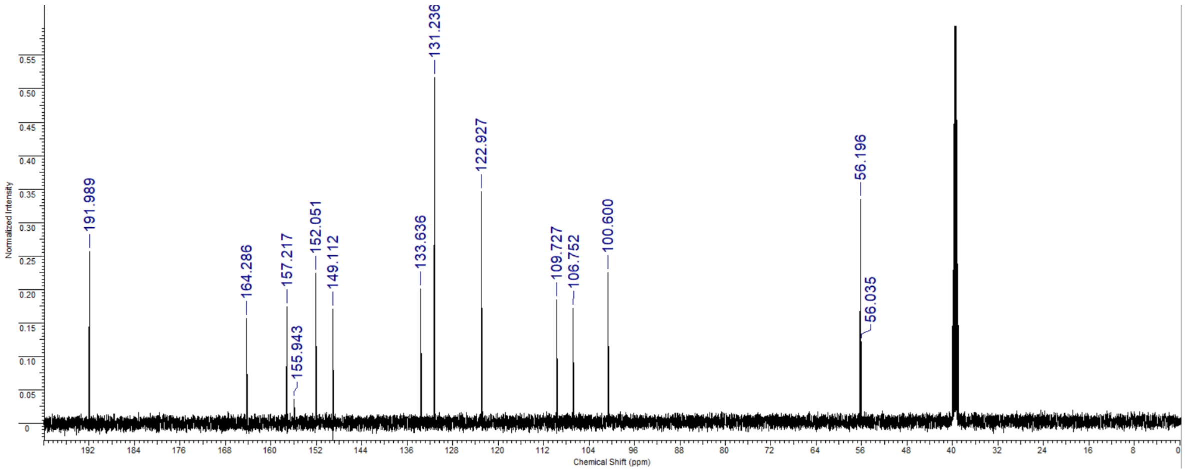

Figure S28. The *^{13}C-NMR* spectrum of Intermediate (1)

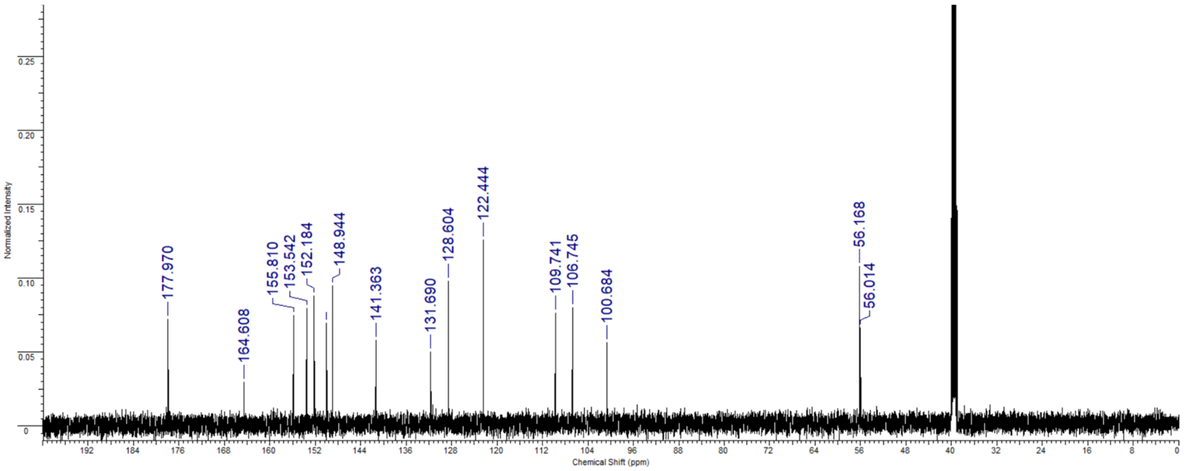

Figure S29. The ^{13}*C-NMR* spectrum of Intermediate (2)

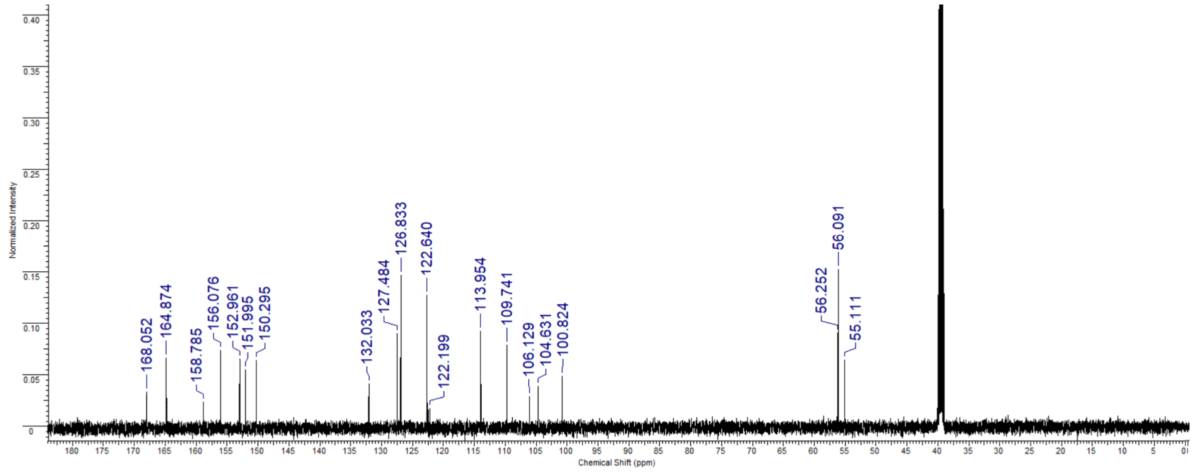

Figure S30. The ^{13}*C-NMR* spectrum of Compound SA01

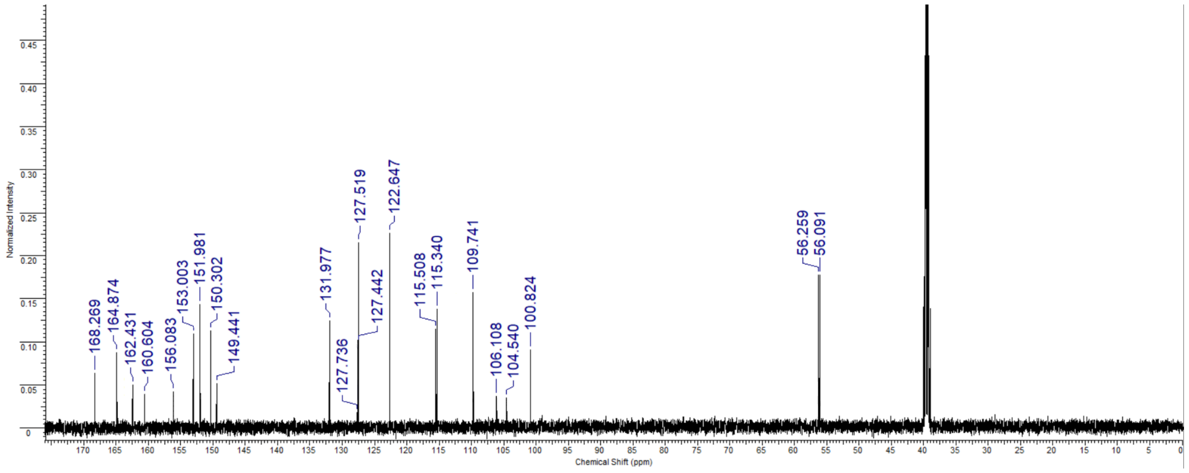

Figure S31. The ^{13}*C-NMR* spectrum of Compound SA02

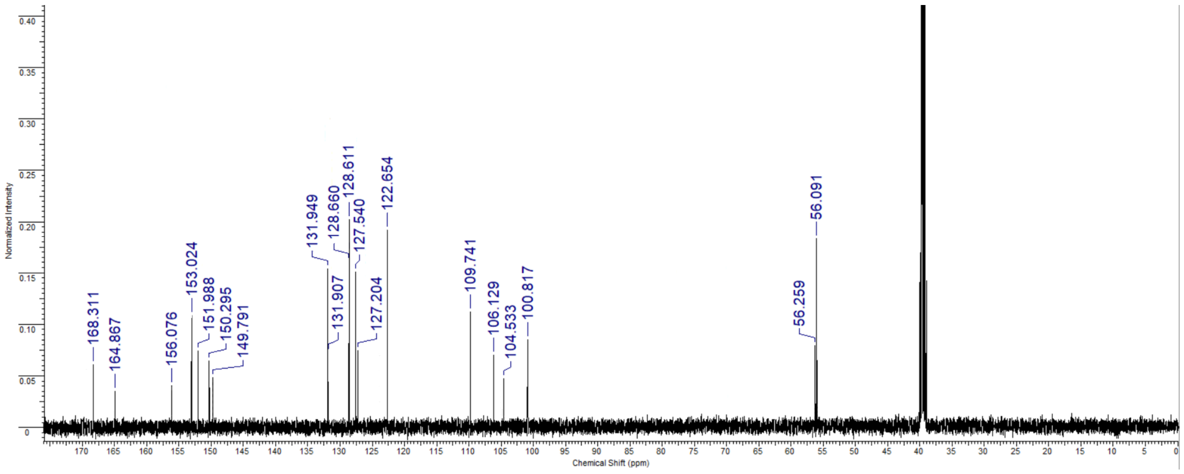

Figure S32. The ^{13}*C-NMR* spectrum of Compound SA03

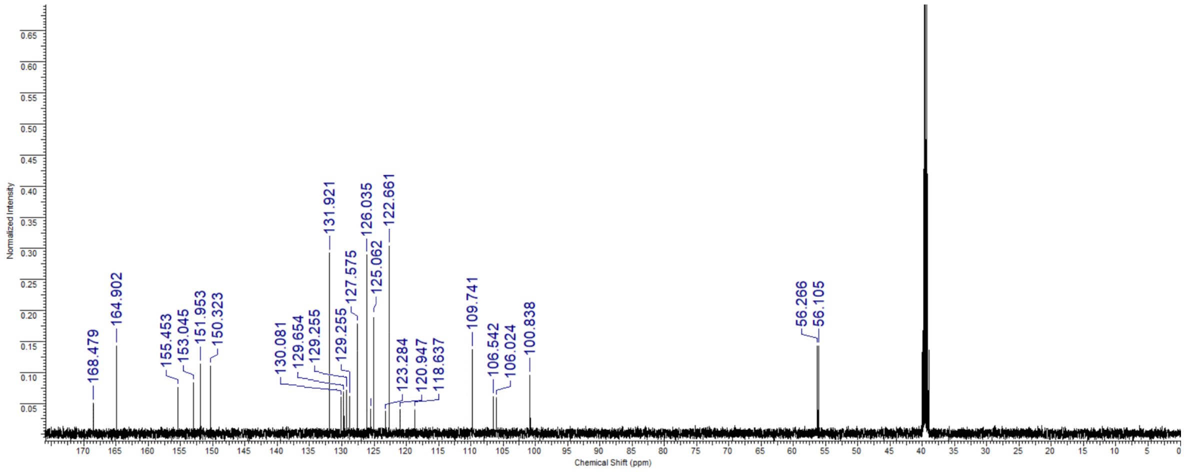

Figure S33. The ^{13}*C-NMR* spectrum of Compound SA04

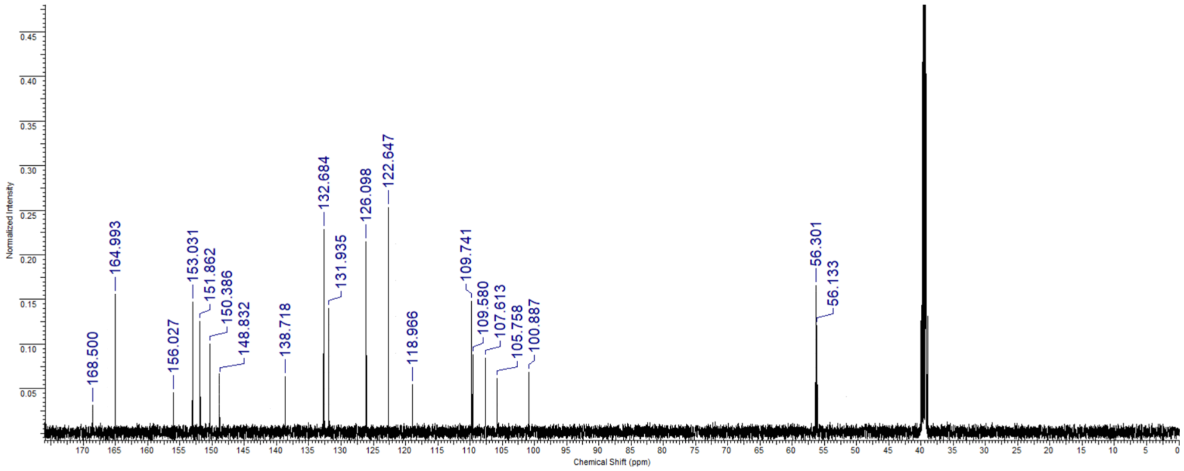

Figure S34. The ^{13}*C-NMR* spectrum of Compound SA05

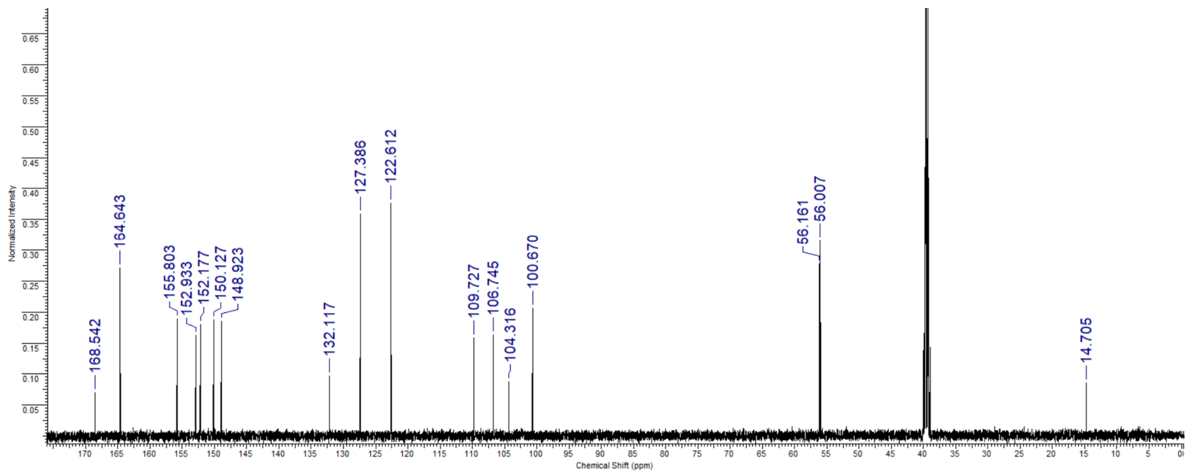

Figure S35. The [13]*C-NMR* spectrum of Compound SA06

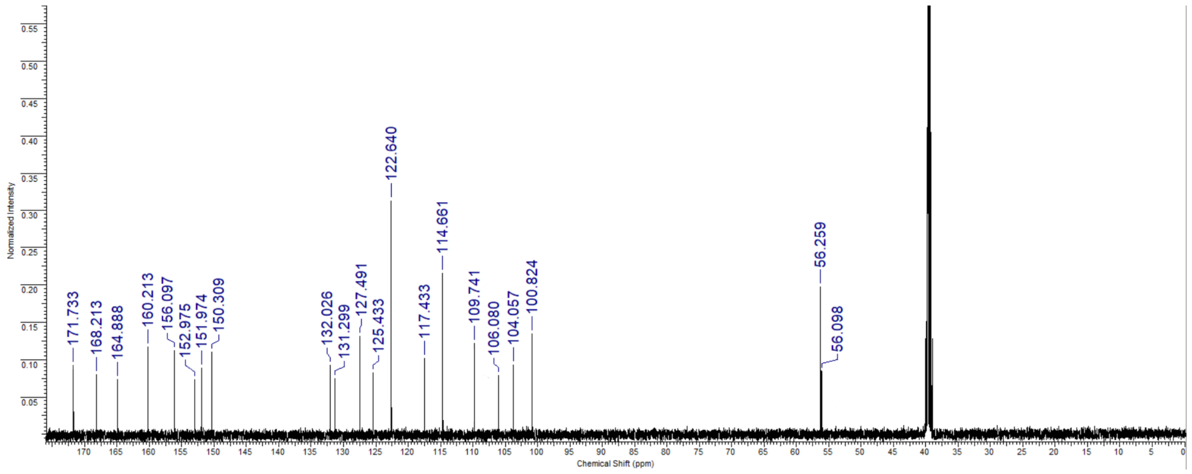

Figure S36. The [13]*C-NMR* spectrum of Compound SA07

1.5. In vitro cytotoxicity evaluation

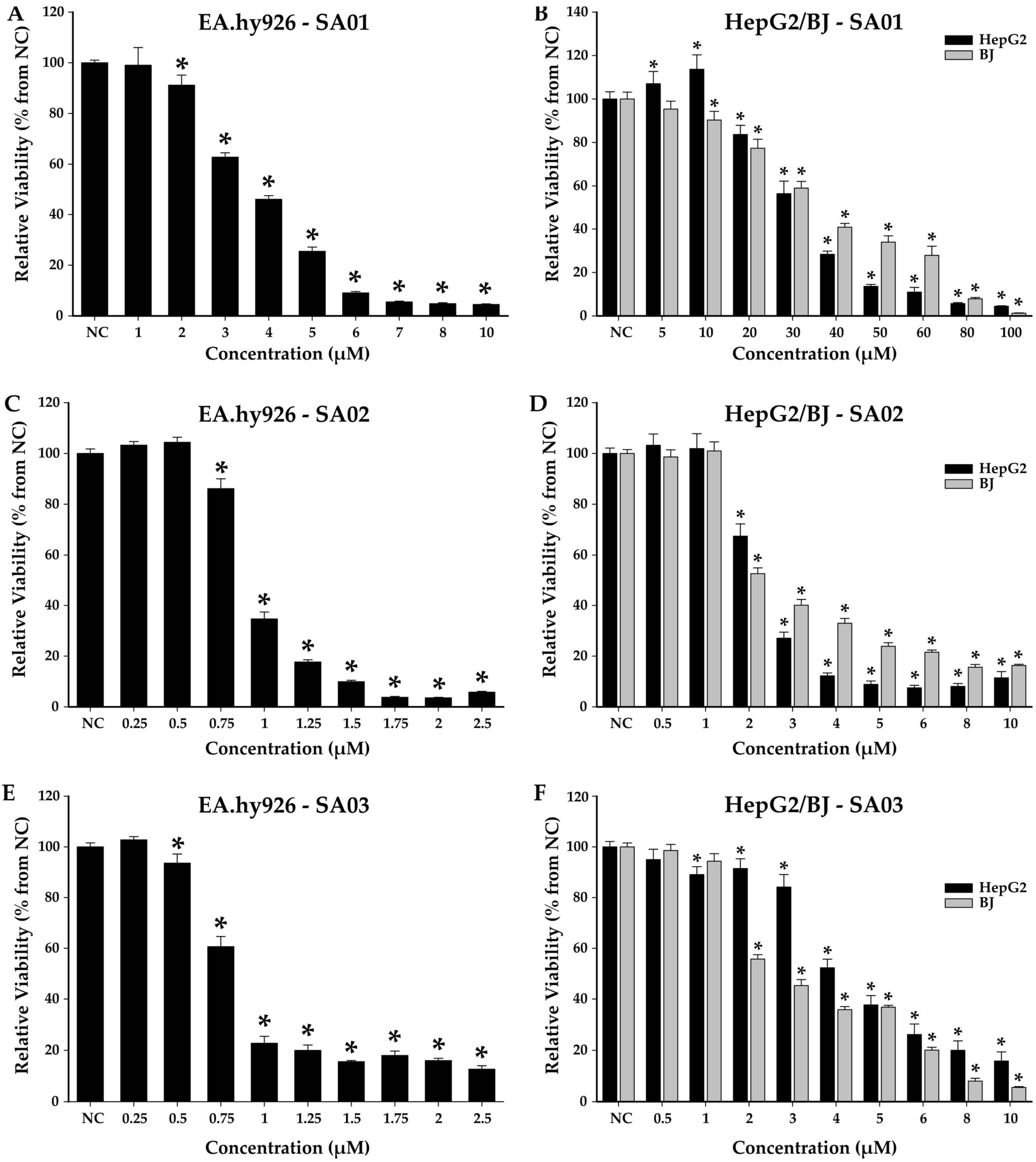

Figure S37. Cytotoxic effect of SA01 (A, B), SA02 (C, D) and SA03 (E, F) after a 48 h exposure of EA.hy926, HepG2 and BJ cells. The results are expressed as relative means ± standard deviations of three biological replicates. Data were expressed as relative values compared to the negative control (NC)(100%). Asterisks (*) indicate significant differences ($p < 0.05$) compared to NC.

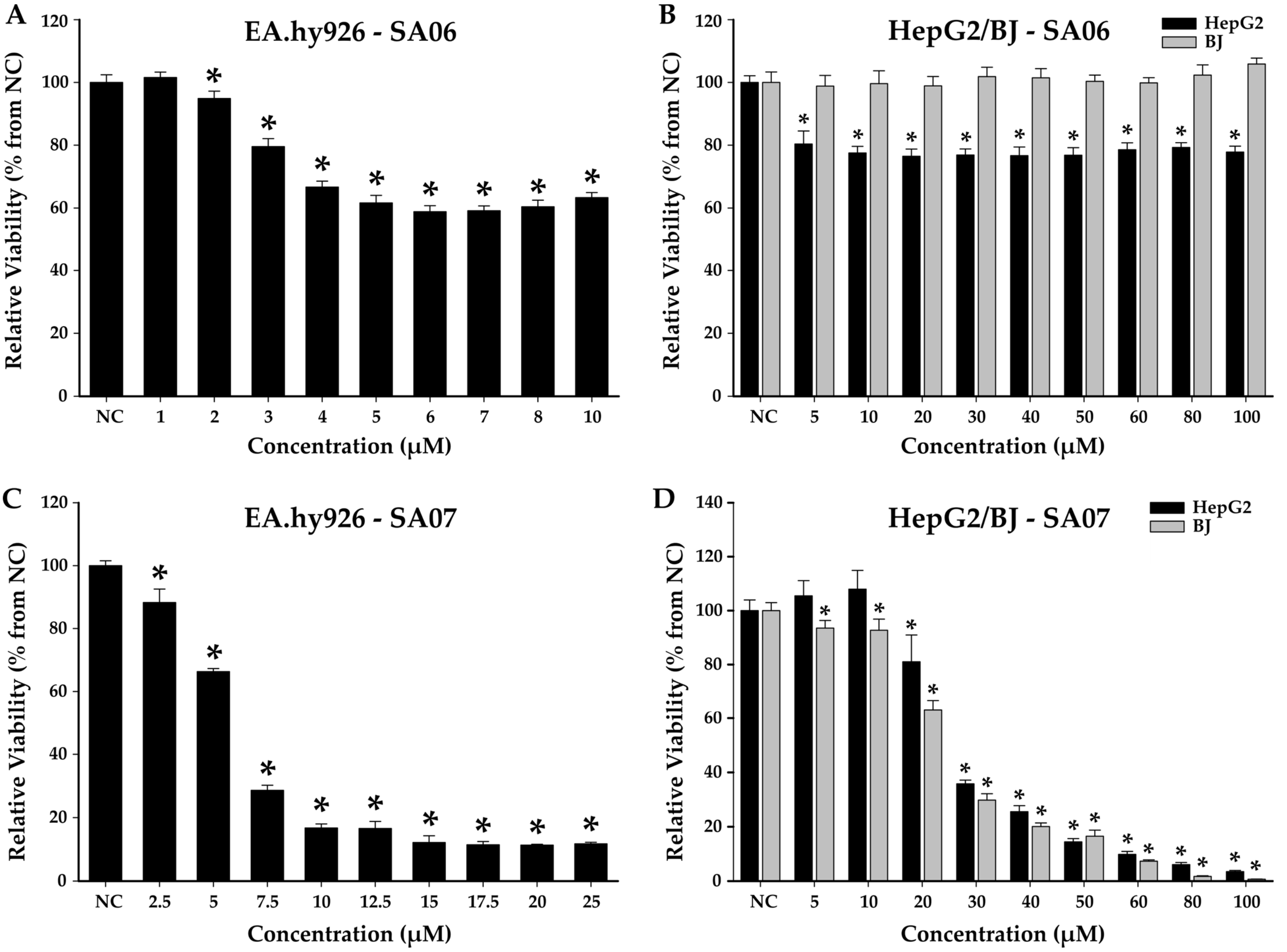

Figure S38. Cytotoxic effect of SA06 (A,B) and SA07 (C,D) after a 48 h exposure of EA.hy926, HepG2 and BJ cells. The results are expressed as relative means ± standard deviations of three biological replicates. Data were expressed as relative values compared to the negative control (NC)(100%). Asterisks (*) indicate significant differences ($p < 0.05$) compared to NC.

1.6. Molecular Dynamics studies

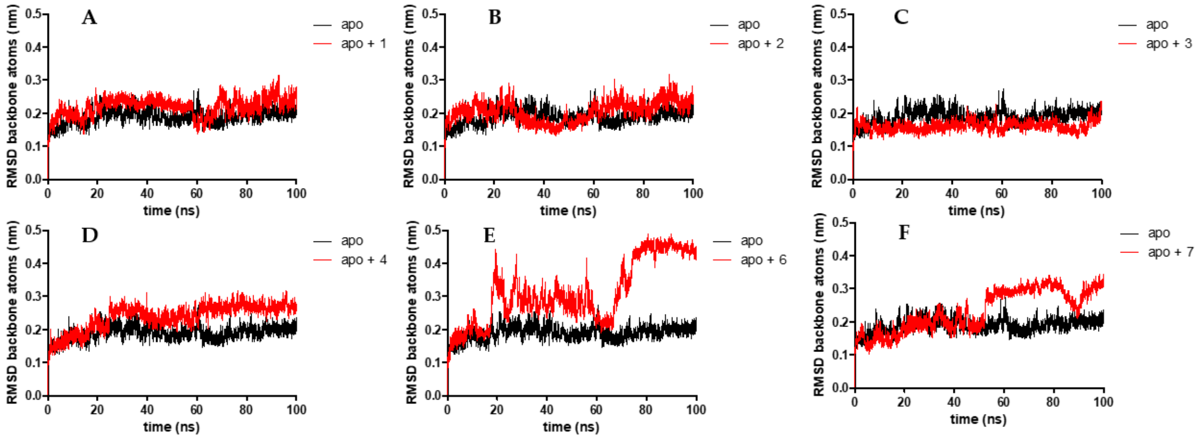

Figure S39. Graphical representation of Root means square deviation (RMSD) of the heavy atoms of VEGFR2 during the 100 ns simulation for SA01 (A); SA02 (B); SA03 (C); SA04 (D); SA06 (E); SA07 (F);

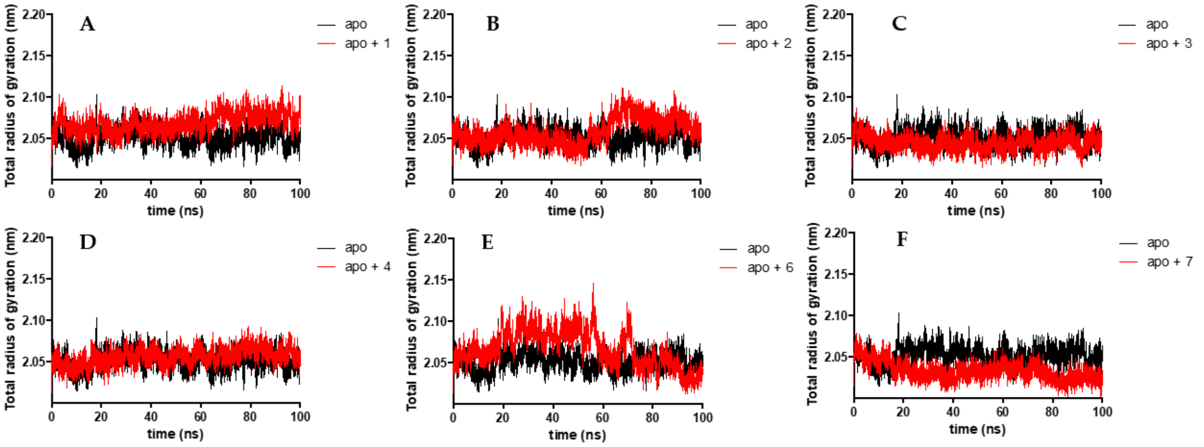

Figure S40. Graphical representation of the radius of gyration during the 100 ns simulation for SA01 (A); SA02 (B); SA03 (C); SA04 (D); SA06 (E); SA07 (F);

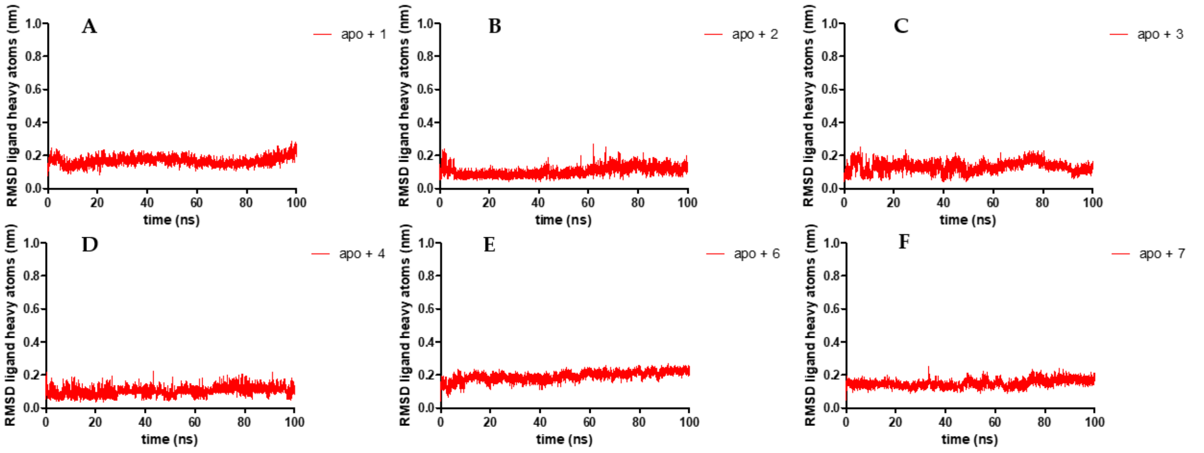

Figure S41. RMSD of ligand's heavy atoms during the 100 ns simulation for SA01 (A); SA02 (B); SA03 (C); SA04 (D); SA06 (E); SA07 (F);

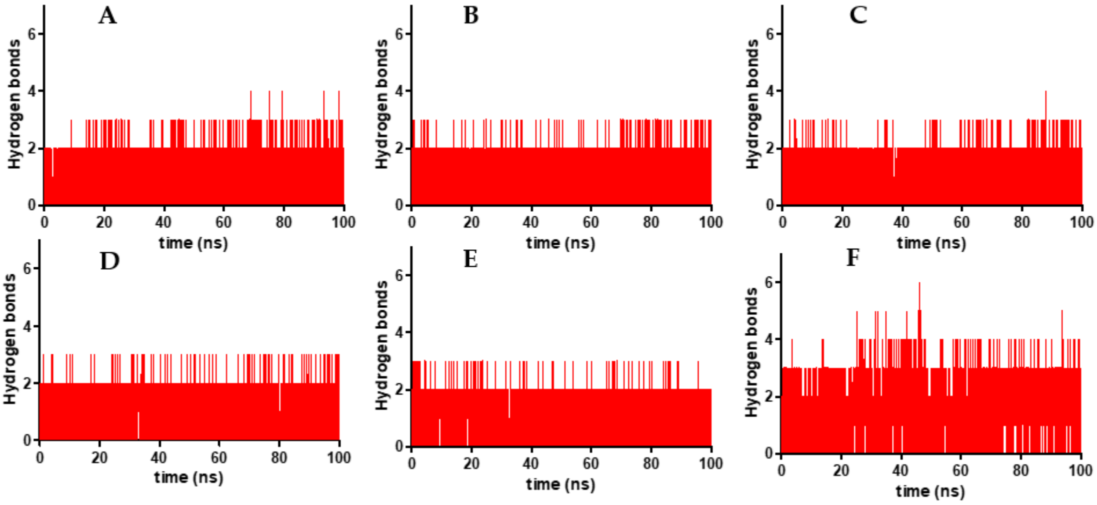

Figure S42. Number of hydrogen bonds encountered during the 100 ns simulation for SA01 (A); SA02 (B); SA03 (C); SA04 (D); SA06 (E); SA07 (F) complex with VEGFR2.

Table S1. Energetic decomposition for the amino-acids found within 5Å from the ligands (kcal/mol)

PDB	SA02	SA03	SA04	SA05	Sorafenib
PRO812	0.00	-0.35	0.00	0.00	0.00
LEU813	-0.31	-0.77	-0.24	0.00	0.00
ASP814	0.14	11.91	0.06	0.16	0.00
LYS838	-0.22	0.00	-0.08	0.00	-0.01
LEU840	-1.00	-0.94	-1.09	-0.86	-1.24
GLY841	-0.26	-0.28	-0.20	-0.26	0.00
VAL848	-0.92	-0.89	-0.75	-0.82	-0.81
GLU850	0.00	0.00	-0.07	0.00	0.00
ALA866	-0.57	-0.61	-0.58	-0.52	-1.09
VAL867	0.00	-0.31	0.00	-0.36	-0.23
LYS868	-1.71	-1.38	-2.43	-1.47	0.67
GLU885	-1.24	-0.51	-0.25	-2.03	-4.20
ILE888	-1.05	-1.38	-1.13	-1.13	-0.59
LEU889	-1.36	-1.44	-1.36	-1.32	-1.44
ILE892	-0.34	-0.38	-0.29	-0.19	-1.03
VAL898	0.00	-0.44	-0.58	0.00	-0.97
VAL899	-1.38	-1.23	-1.36	-1.47	-1.04
VAL914	-0.44	-0.47	-0.39	-0.54	-0.42
VAL916	-1.06	-1.16	-1.23	-1.19	-1.14
GLU917	-1.33	-1.32	-0.91	-1.20	-2.39
PHE918	-1.80	-1.82	-1.82	-1.76	-1.25
CYS919	-1.87	-1.73	-1.70	-1.75	-1.66
LYS920	-0.18	0.02	-0.03	-0.14	-0.19
PHE921	-0.08	-0.12	-0.12	-0.06	-0.11
GLY922	-0.29	-0.19	-0.26	-0.18	-0.35
ASN923	1.03	0.73	0.72	0.62	-0.08
LEU1019	-0.47	-0.16	-0.50	-0.62	-0.71
CYS1024	-0.61	-0.61	-0.84	0.00	-0.35
ILE1025	0.60	0.87	0.79	-0.08	0.78
HIS1026	0.39	0.35	0.19	-0.28	-0.43
ARG1027	-0.34	-1.51	0.20	0.39	0.00
LEU1035	-1.49	-1.41	-1.36	-1.34	-1.08
ILE1044	-0.48	-0.33	-0.39	-0.45	-0.51
CYS1045	-1.89	-1.99	-1.97	-1.68	-2.69
ASP1046	-0.31	-0.74	-0.56	-0.51	-2.11
PHE1047	-1.47	-1.19	-1.33	-1.72	-2.22
ALA1050	0.00	0.04	0.00	0.00	0.00

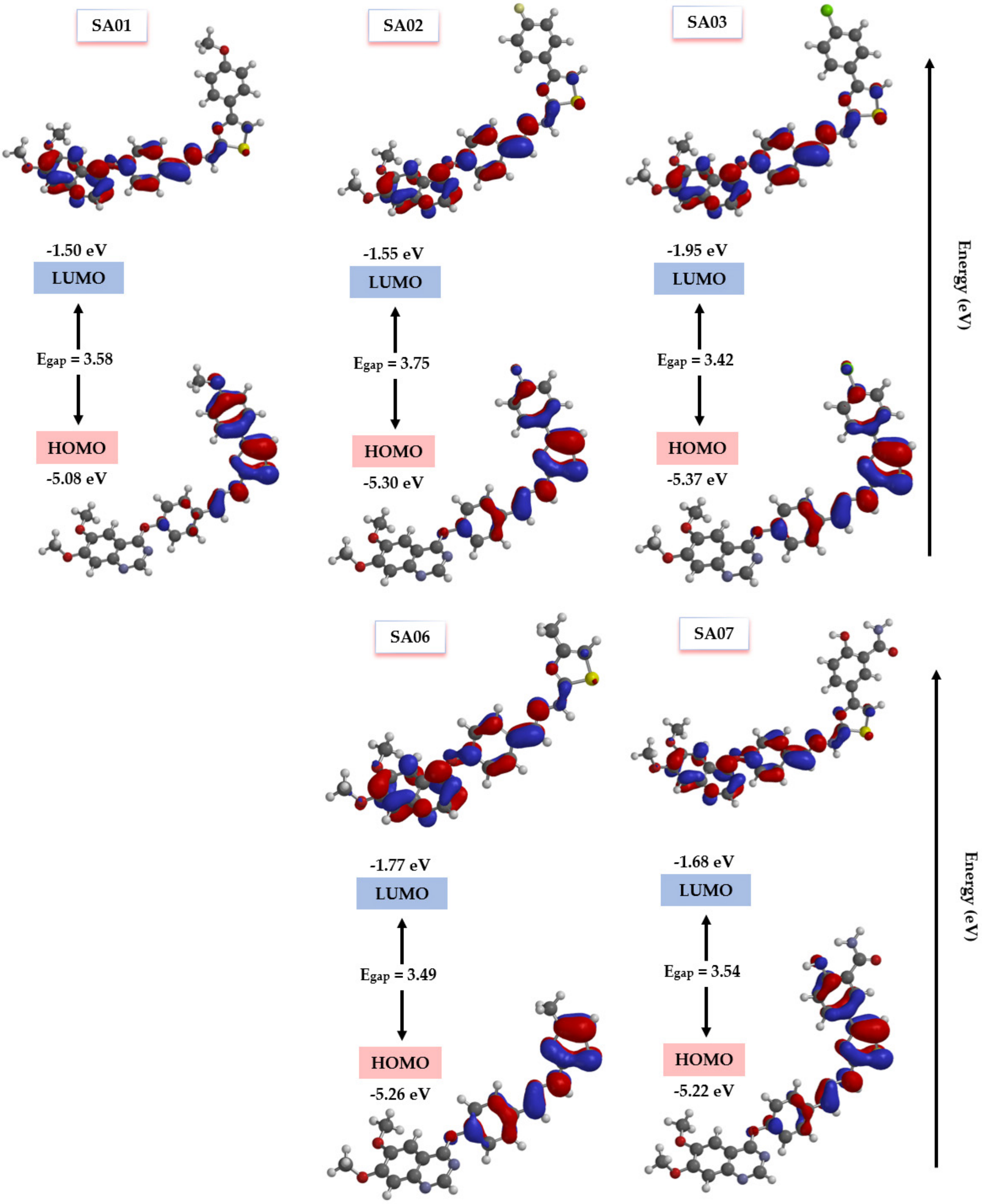

Figure S43. Graphical representation of molecular orbitals surface distribution and energy levels of HOMO and LUMO of the studied compounds **SA01-SA03, SA06-SA07** at the B3LYB/6-311G++(d,p) level;

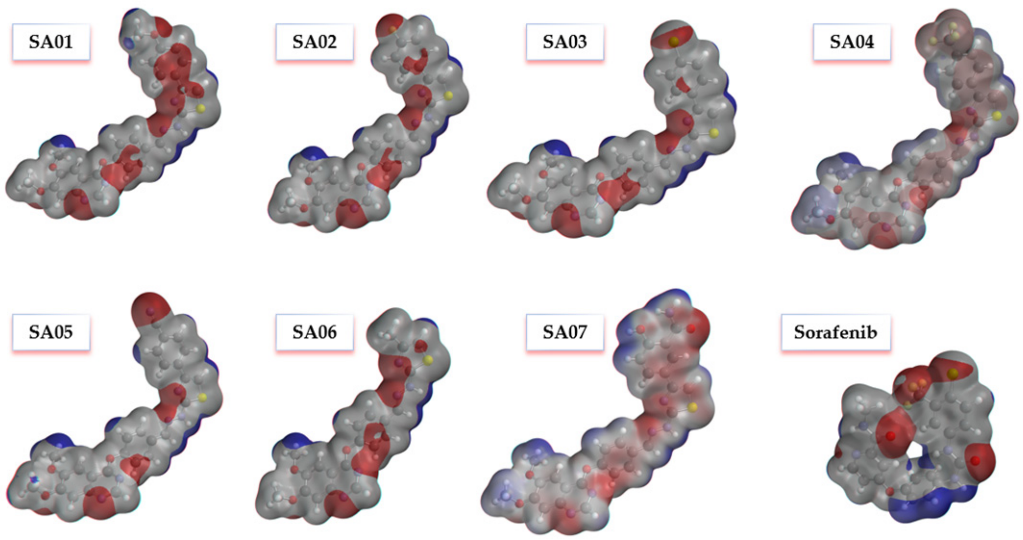

Figure S44. Graphical representation of the molecular electrostatic map (MEP) of **SA01-SA07** and reference drug **sorafenib**

Table S2. *In silico* ADME profile and drug-likeness of the **SA01-SA07** series & **Sorafenib** generated by SwissADME

ADME parameters	SA01	SA02	SA03	SA04	SA05	SA06	SA07	Sorafenib
Physicochemical properties								
Molecular Weight (g/mol)	513.57	501.53	517.99	551.54	508.55	421.47	542.57	464.82
Num. Heavy Atoms	37	36	36	39	37	30	39	32
Num. Ar. Heavy Atoms	27	27	27	27	27	21	27	18
Fraction Csp^3	0.11	0.08	0.08	0.11	0.07	0.14	0.07	0.10
Num. Rotatable Bonds	9	8	8	9	8	7	9	9
Num. H-bonds Acceptors	8	8	7	10	8	7	9	7
Num. H-bonds Donnors	1	1	1	1	1	1	3	3
[1]TPSA ($Å^2$)	128.22	118.99	118.99	118.99	142.78	118.99	182.31	92.35
Lipophilicity								
Log $P_{o/w}$ (XLOGP3)	5.96	6.09	6.62	6.87	5.71	4.73	5.06	4.07
Log $P_{o/w}$ (MLOGP)	2.46	3.4	3.5	3.53	2.39	2.18	1.53	2.91
Water Solubility								
Log S (ESOL)	-6.72	-6.81	-7.25	-7.51	-6.6	-5.49	-6.31	-5.11
[2]Class	Poorly soluble	Poorly soluble	Poorly soluble	Poorly soluble	Poorly soluble	Moderately soluble	Poorly soluble	Moderately soluble
Pharmakocinetics								
[3]GI absorbtion	Low	Low	Low	Low	Low	High	Low	Low
[4]BBB permeant	No	No	No	No	No	No	No	No
[5]P-gp substrate	No	No	No	No	No	No	No	No
CYP1A2 inhibitor	No	No	No	No	No	Yes	No	Yes
CYP2C19 inhibitor	Yes	No	No	Yes	No	Yes	Yes	Yes
CYP2C9 inhibitor	Yes	Yes	Yes	Yes	Yes	Yes	Yes	Yes
CYP2D6 inhibitor	Yes	No	No	No	No	No	No	Yes
CYP3A4 inhibitor	Yes	Yes	Yes	Yes	Yes	Yes	Yes	Yes

Drug-likeness								
Lipinski Violations	1	1	1	1	1	0	2	0
Veber Violations	0	0	0	0	1	0	1	0

[1]Topological polar surface area, [2]Log S scale (insoluble < -10 < poorly < -6 < moderately <-4 soluble < -2 < very < 0 < highly); [3] gastrointestinal; [4] blood-brain barriers; [5] p-glycoprotein

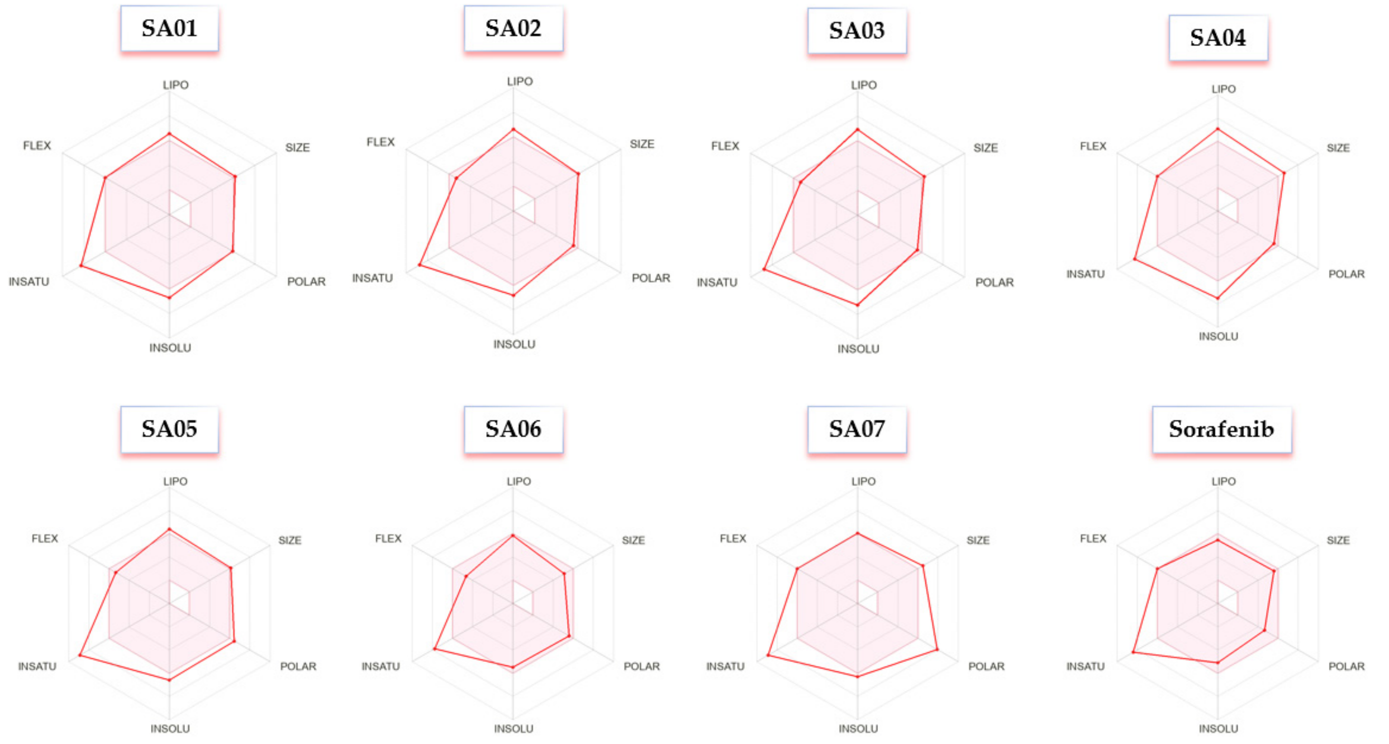

Figure S45. The bioavailability radar for the **SA01-SA07** series & **Sorafenib** generated by SwissADME (The red zone represents the suitable range for the parameters for oral absorption); LIPO (lipophilicity, -7 < XLOGP3 < +5.0), SIZE (Molecular Weight g/mol, 150 < SIZE <500; POLAR (Polarity, 20 Å² < POLAR < 130 Å²); INSOLU (insolubility; -6 < Log S (ESOL) < 0); INSATU (Insaturation; 0.25 < Fraction Csp³ < 1); FLEX (Flexibility, 0 < Num. of rotatable bonds < 9).
